# A Novel C_2_H_2_ Transcription Factor that Regulates *gliA* Expression Interdependently with GliZ in *Aspergillus fumigatus*

**DOI:** 10.1371/journal.pgen.1004336

**Published:** 2014-05-01

**Authors:** Taylor J. Schoberle, C. Kim Nguyen-Coleman, Jennifer Herold, Ally Yang, Matt Weirauch, Timothy R. Hughes, John S. McMurray, Gregory S. May

**Affiliations:** 1 The University of Texas Graduate School of Biomedical Sciences at Houston, The University of Texas MD Anderson Cancer Center, Houston, Texas, United States of America; 2 Microbiology and Molecular Genetics, Division of Pathology and Laboratory Medicine, The University of Texas MD Anderson Cancer Center, Houston, Texas, United States of America; 3 Banting and Best Department of Medical Research, Donnelly Centre, and Department of Molecular Genetics, University of Toronto, Toronto, Ontario, Canada; 4 Department of Experimental Therapeutics, Division of Cancer Medicine, The University of Texas MD Anderson Cancer Center, Houston, Texas, United States of America; 5 Program in Genes and Development, The University of Texas MD Anderson Cancer Center, Houston, Texas, United States of America; University College Dublin, Ireland

## Abstract

Secondary metabolites are produced by numerous organisms and can either be beneficial, benign, or harmful to humans. Genes involved in the synthesis and transport of these secondary metabolites are frequently found in gene clusters, which are often coordinately regulated, being almost exclusively dependent on transcription factors that are located within the clusters themselves. Gliotoxin, which is produced by a variety of *Aspergillus* species, *Trichoderma* species, and *Penicillium* species, exhibits immunosuppressive properties and has therefore been the subject of research for many laboratories. There have been a few proteins shown to regulate the gliotoxin cluster, most notably GliZ, a Zn_2_Cys_6_ binuclear finger transcription factor that lies within the cluster, and LaeA, a putative methyltransferase that globally regulates secondary metabolism clusters within numerous fungal species. Using a high-copy inducer screen in *A. fumigatus*, our lab has identified a novel C_2_H_2_ transcription factor, which plays an important role in regulating the gliotoxin biosynthetic cluster. This transcription factor, named GipA, induces gliotoxin production when present in extra copies. Furthermore, loss of *gipA* reduces gliotoxin production significantly. Through protein binding microarray and mutagenesis, we have identified a DNA binding site recognized by GipA that is in extremely close proximity to a potential GliZ DNA binding site in the 5′ untranslated region of *gliA*, which encodes an efflux pump within the gliotoxin cluster. Not surprisingly, GliZ and GipA appear to work in an interdependent fashion to positively control *gliA* expression.

## Author Summary

Fungal infections cause severe problems for immune-compromised individuals. Current antifungal treatment is problematic, as some therapies are toxic to humans and others are not highly effective. These fungal infections also burden hospitals, as costs to treat and prevent such disease runs high. *Aspergillus fumigatus* is the most common cause of fungal infections worldwide. *A. fumigatus* produces a variety of toxins that aid the fungus in survival both in the environment and within host systems. Genes involved in producing such toxins are often found in clusters within the genome, being almost exclusively dependent on transcription factors located within the clusters. Gliotoxin, one such toxin, is known to negatively affect immune cell function. Although gliotoxin has been studied extensively, information is still lacking with regards to regulation of gliotoxin biosynthesis. Our lab has discovered a novel C_2_H_2_ transcription factor, GipA, which plays an important role in gliotoxin production. Not only does GipA enhance gliotoxin production when over-expressed, but loss of GipA causes a significant reduction in gliotoxin production. As this gene is not located within the gliotoxin cluster, understanding its mode of action and upstream partners could shed light on toxin production in general and lead to better, more effective antifungal therapies.

## Introduction

Secondary metabolites are small, low-molecular mass molecules made by numerous organisms that are not essential for normal growth, but can play important roles in defense or signaling [1]–[4]. They can be benign in nature, such as pigments or molecules used in interspecies communication, but they can also be malignant, exhibiting antimicrobial or toxic activities to eliminate competing organisms [5], [6]. Some of these compounds have been exploited by scientists because of their potential benefit to humans. For example, penicillin, produced by *P. chrysogenum*, is used as an antibiotic and lovastatin, produced by *A. terreus*, reduces cholesterol [7]. While some of these secondary metabolites benefit humans, others cause harm. Aflatoxin, produced by *A. flavus*, is carcinogenic, and gliotoxin, produced by *A. fumigatus*, exhibits immunosuppressive properties [4], [8]–[10]. Genes involved in secondary metabolite production are frequently found as clusters, which are often located in subtelomeric positions [2]–[5], [11]. Furthermore, these clusters are often coordinately regulated, being almost completely dependent on induction by transcription factors located within the clusters themselves [3]–[5], [12], [13].

Zinc binuclear (Zn(II)_2_Cys_6_) transcription factors are uniquely found in fungi and represent the most common type of regulators located within these clusters [3], [4], [14], [15]. AflR, a Zn_2_Cys_6_ transcription factor, is located within the aflatoxin/sterigmatocystin gene cluster and is required for production of both metabolites [3], [4], [10]. Zn_2_Cys_6_ transcription factors, like *aflR*, generally recognize and bind as homodimers to palindromic sequence motifs, such as CGG(N_x_)CCG [14]–[17]. Interestingly, although the palindromic sequences of these binding motifs can be similar or identical for multiple Zn_2_Cys_6_ transcription factors, the length and base composition of the linker sequence is highly variable. Therefore, this linker sequence greatly contributes to the specificity of binding for each individual transcription factor [15], [17], [18]. Although gene clusters are often coordinately regulated by the cluster-specific transcription factor, some members can be independently regulated. For example, *gliT* encodes an oxidoreductase of the gliotoxin biosynthetic cluster, which is required for self-protection against gliotoxin. Even though *gliT* expression is decreased when the Zn_2_Cys_6_ transcription factor, *gliZ*, is deleted, exogenous gliotoxin induces the expression of *gliT*, even in a *ΔgliZ* background [4], [19].

Aside from pathway-specific transcription factors that reside within the cluster, there are numerous other regulatory elements that affect the expression of secondary metabolite clusters. Nutritional and environmental factors, as well as developmental processes, have been shown to affect secondary metabolite production in multiple fungal species [3]–[5]. For instance, penicillin production in *A. nidulans* is repressed in the presence of glucose, a phenomenon termed carbon catabolite repression [8], [20]. Secondary metabolite repression also occurs in response to nitrogen source, which involves AreA, the global positive regulator of nitrogen metabolite repression. Indeed, in *A. nidulans*, sterigmatocystin production is repressed in the presence of ammonium and induced when nitrate is the sole nitrogen source [2], [6], [21]. This type of regulation is not seen in all fungi, as carbon and nitrogen source have the opposite effect in *A. parasiticus*, where growth in the presence of glucose or ammonium results in higher levels of aflatoxin production [2], [6], [21].

*A. fumigatus*, the leading cause of mold infections worldwide, is an opportunistic pathogen that causes severe problems in immune-compromised populations [9], [22]. These populations include: AIDS patients, cancer patients receiving chemotherapy, solid organ transplant/skin graft patients and victims of chronic granulomatous disease [12], [23]–[25]. One of the most studied secondary metabolites produced by *A. fumigatus* is gliotoxin, which is also produced by several other *Aspergillus* species, *Trichoderma* species, and *Penicillium* species [13], [26]–[28]. Gliotoxin is a member of the epidithiodioxopiperazine (ETP) class of toxins, which are characterized by a disulfide bridge across a piperazine ring [13], [23]–[27], [29]–[33]. The oxidized form of gliotoxin travels into host immune cells where it is able to affect cellular functions essential to the immune response. These include impediment of phagocytosis and NF-κB activation, as well as induction of apoptosis [23], [25], [26], [29], [32], [33]. As with other secondary metabolites, most of the genes responsible for the production and transport of gliotoxin exist within a gene cluster. The gliotoxin biosynthesis cluster was first identified based on its homology to the sirodesmin PL biosynthesis gene cluster in the ascomycete *Leptosphaeria maculans* [13], [27], [34], [35].

Within this cluster lies a Zn_2_Cys_6_ binuclear finger transcription factor, GliZ, thought to be responsible for general gliotoxin induction and regulation. Indeed, over-expression of *gliZ* leads to an increase in gliotoxin production and deletion of *gliZ* results in a loss in gliotoxin production [12], [26], [28]. A DNA binding site has been proposed for GliZ (TCGGN_3_CCGA), but has not been experimentally proven. This site is present within the promoter region of every gene within the gliotoxin cluster, except *gliZ* and *gliA*, which encodes an efflux pump within the cluster [14]. Gliotoxin itself positively regulates expression of the genes within the gliotoxin cluster, as deletion of *gliP*, the non-ribosomal peptide synthetase (NRPS) required for the first step in the synthesis of gliotoxin, virtually eliminates expression of the other genes in the cluster. This loss in gene expression can be reversed by the addition of exogenous gliotoxin to culture medium [19], [26], [30]. Interestingly, *gliT*, encoding an oxidoreductase required for resistance of the fungus to gliotoxin, is induced by exogenous gliotoxin even in the absence of *gliZ* [19]. This demonstrates regulation of genes within the cluster that are independent of the coordinate regulation by GliZ. LaeA, a global regulator of secondary metabolism, has also been shown to regulate the gliotoxin cluster, as gliotoxin is among the secondary metabolites that are lost with deletion of *laeA* [36]–[38]. Furthermore, loss of *vel1* in *T. virens* (homologous to VeA in *Aspergilli*) results in a loss in gliotoxin production [39]. This is not surprising, since VeA, VelB, and LaeA form a heterotrimeric complex that regulates secondary metabolism in several fungal species [2], [25], [36], [37], [40]. RsmA, a bZIP transcription factor, positively regulates the gliotoxin cluster through LaeA and GliZ, as loss of either protein abolishes the inducing effects of RsmA over-expression [41]. Map kinase signaling is another element that is important for gliotoxin production, as a strain lacking *mpkA*, the map kinase in the cell wall integrity pathway, is significantly reduced in gliotoxin production [42]. Nutrient starvation, murine infection, and exposure of germlings to neutrophils have also been shown to up-regulate the gliotoxin biosynthesis cluster through microarray analysis [11], [26].

At this time, information is still lacking as to what proteins and transcriptional regulators are responsible for differential expression of the gliotoxin cluster. We have identified a novel C_2_H_2_ transcription factor, *gipA*, which plays a role in regulating the gliotoxin biosynthesis gene cluster. This protein was identified using a high-copy inducer screen and has not been discussed in detail before now. High-copy expression of *gipA* results in an increase in transcription of multiple genes within the gliotoxin cluster. Conversely, loss of *gipA* negatively affects expression of multiple genes within the gliotoxin cluster. In addition, this C_2_H_2_ transcription factor specifically regulates *gliA* expression through a binding site we have identified. Here, we propose a model for *gliA* expression that involves both GliZ and GipA. Our aim was to identify a novel protein that is involved in gliotoxin production, which we achieved through the discovery of GipA.

## Results

### High-Copy Inducer Screen Identifies a Novel Protein That Induces Expression of a *gliA^P^-lacZ* Cassette

To identify genes that regulate the gliotoxin biosynthesis cluster, we performed a high-copy inducer screen. Our goal was to identify genes that, when present in extra copies, induce the gliotoxin biosynthesis cluster in repressing conditions. We used a LacZ reporter system, under the control of the *gliA* promoter (identified as GL in strain names). GliA encodes an efflux pump within the gliotoxin cluster that is thought to be involved in the transport of gliotoxin out of the fungal cell [13], [27]. Although GliA has not been shown to be essential to gliotoxin transport in *A. fumigatus*, expression of *gliA* in a mutant strain of *L. maculans* provided protection against gliotoxin, but not sirodesmin [27]. We chose *gliA* for several reasons. First, it has been shown that expression of *gliA* peaks when the amount of gliotoxin in surrounding medium is maximal [13], [27]. Second, experiments in our lab revealed that *gliA* is induced within 30 min of *A. fumigatus* germlings being exposed to human neutrophils, while nutritional induction of the gliotoxin cluster takes 24–48 hrs (data not shown). These data led us to conclude that *gliA* expression would be a good indicator of gliotoxin cluster expression, as well as gliotoxin production.

**Figure 1:**
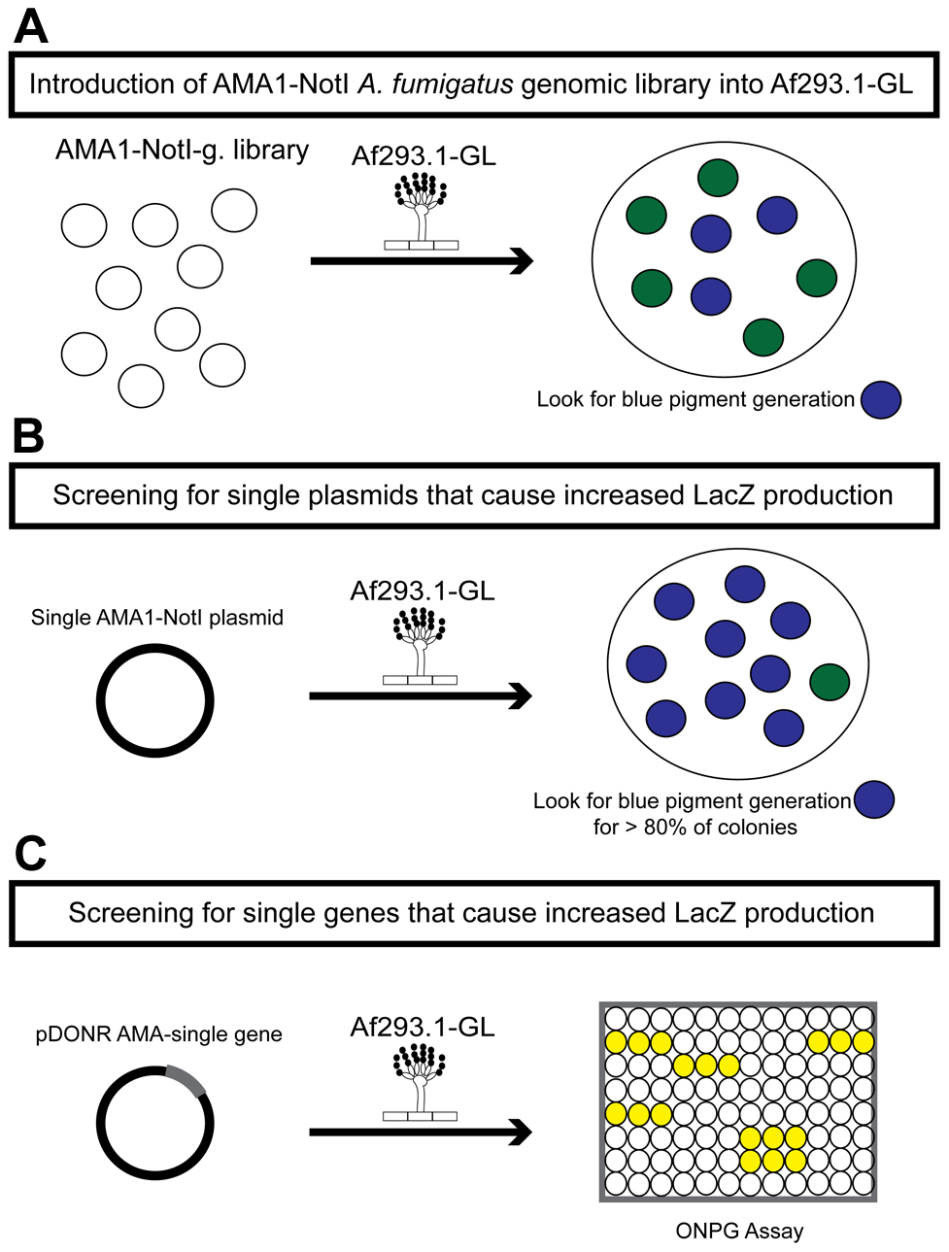
Schematic of the three rounds of the high-copy inducer screen. (a) Round one: transformation of the genomic library into Af293.1-GL. (b) Round two: transformation of single plasmids from the genomic library into Af293.1-GL. (c) Round three: transformation of single genes into Af293.1-GL.

The first round of the high-copy inducer screen involved transforming an AMA1-NotI *A. fumigatus* genomic library [43] into Af293.1-GL (Fig. 1a). AMA1 stands for autonomous maintenance in Aspergillus and was first discovered and isolated from *A. nidulans* [44], [45]. Due to the presence of this element, the *A. fumigatus* library plasmids replicate autonomously upon transformation. This feature allows for easy recovery of the plasmids from the fungal genome by transformation of the genomic DNA into bacteria [46], [47]. Furthermore, this screen is a high-copy inducer screen, because there are on average 10 to 30 copies of any given AMA1-containing plasmid per genome [44], [45]. We grew transformants on gliotoxin-repressing medium with X-gal and screened for colonies that produced a blue pigment. This indicated that the AMA1-NotI plasmid within the genome was inducing gliotoxin cluster expression in repressing conditions. For the second round of the high-copy inducer screen, we transformed individual plasmids, isolated in the first round of the genetic screen, into Af293.1-GL (Fig. 1b). We transformed individual vectors to be sure that the effect we observed from the first round was the result of only one plasmid and not multiple plasmids. To measure LacZ levels in a more quantitative manner, we isolated total protein from transformants and measured β-galactosidase activity. We transformed a pDONR AMA empty vector (AMA.GL) and pDONR AMA-*gliZ* (AMA-gliZ.GL) as a negative control and positive control, respectively. The third round of the high-copy inducer screen entailed isolating the individual genes from each AMA1-NotI library plasmid and transforming them into Af293.1-GL (Fig. 1c). For this round, we only measured β-galactosidase activity.

**Figure 2:**
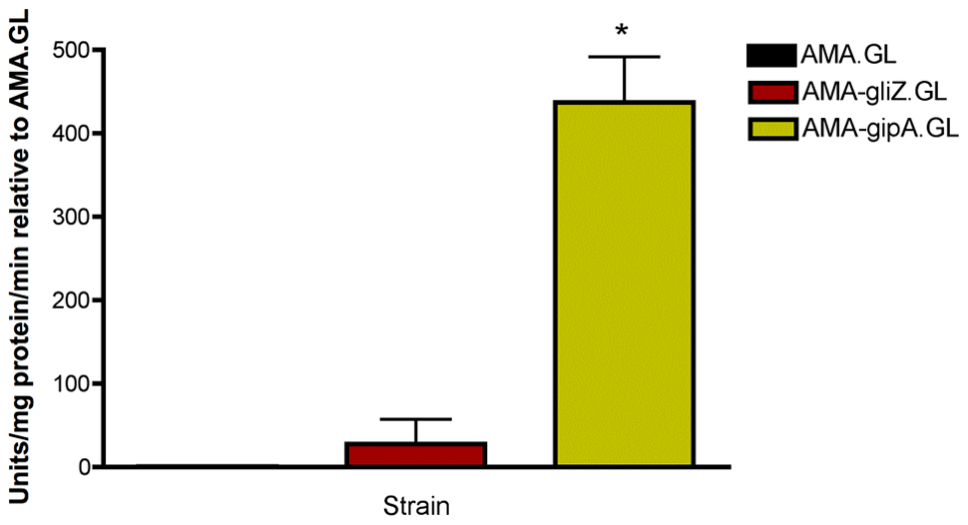
β-galactosidase assays for *gliZ* and *gipA* high-copy expression strains. Data is presented as fold-change relative to AMA.GL. The data presented is an average of three biological replicates. The asterisk (*) indicates a statistically significant difference (p-value<0.05), compared to AMA.GL, calculated by one-way ANOVA and Tukey comparison test.

The gene that induced *lacZ* the most was a C_2_H_2_ transcription factor (AFUA_6G01910), which we have designated *gipA* for gliotoxin inducing protein. High-copy expression of *gipA* in the Af293.1-GL background (AMA-gipA.GL) induces a 400-fold increase in β-galactosidase activity, compared to the empty vector control (AMA.GL) (Fig. 2). The level of β-galactosidase activity in our positive control, AMA-gliZ.GL, was almost 30-fold higher than the empty vector control, AMA.GL. These data indicate that GipA positively regulates *gliA* expression, similar to GliZ.

### *gipA* Is a C_2_H_2_ Transcription Factor with an Unusually Long 5′ UTR

**Figure 3:**
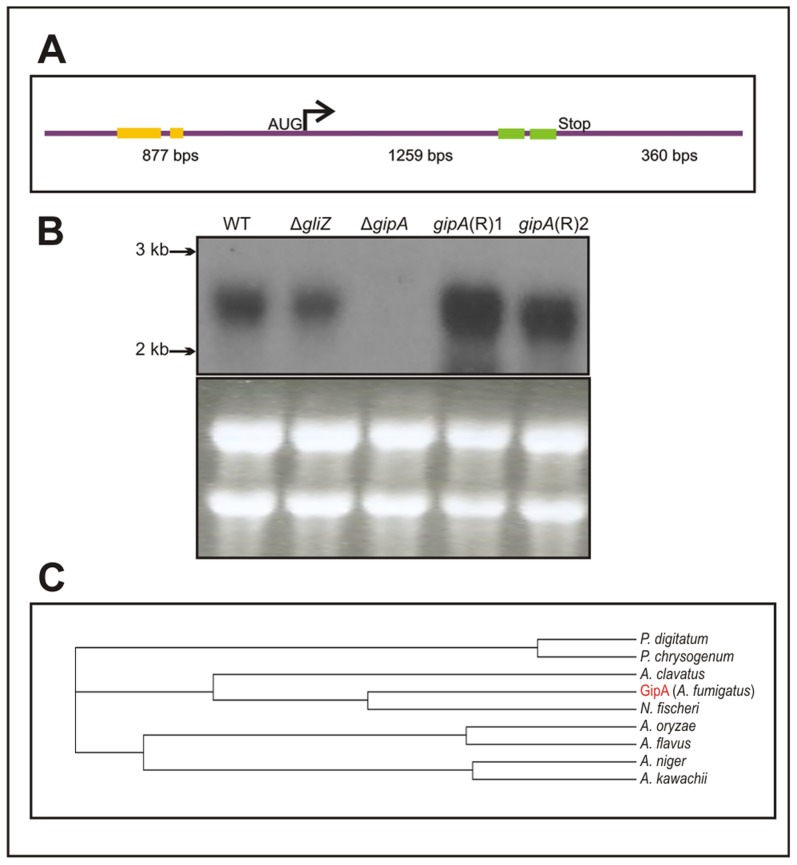
Characterization of *gipA* cDNA. (a) Schematic of cDNA size and composition. Yellow bars signify μORFs and the green bars display the two zinc finger domains. The size indicated for the coding region includes the one intron. (b) Northern hybridization of *gipA* from total RNA. WT is wild-type (Af1160) and (R) signifies complemented strains. (c) Cladogram of GipA homologues. *N. fischeri* is *Neosartorya fischeri*.

The predicted sequence of *gipA* contains one intron and two C_2_H_2_ regions at the 3′ end (Fig. 3a). The two C_2_H_2_ regions are canonical, the first being X_2_-C-X_2_-C-X_12_-H-X_3_-H and the second being X_2_-C-X_2_-C-X_12_-H-X_5_-C, which is a natural variant [48], [49]. The 5′ untranslated region (UTR) of *gipA* appears to be unusually long (877 bp) and contains three μORFs, which indicates that *gipA* may be under post-transcriptional control due to regulation of translational efficiency and mRNA stability. Northern analysis was consistent with cDNA predictions (Fig. 3b). Sequence similarity searches indicated that proteins homologous to GipA are present in only a limited number of species, the closest being a C_2_H_2_ transcription factor in *N. fischeri*, a close relative to *A. fumigatus* (Fig. 3c). The rest of the homologous proteins were from other *Aspergillus* species and a few *Penicillium* species, which suggests that GipA is not highly conserved at the primary sequence. For proteins, primary sequences evolve and change much more rapidly than do the tertiary structures. There have been numerous examples where two proteins have low sequence similarity, but once crystallized, exhibit almost identical folding patterns and subsequently share similar functions [50]. For example, Gcn4 of *Saccharomyces cerevisiae* and CpcA of *A. niger* share a 35% identity, yet they both function in amino acid biosynthesis. Furthermore, CpcA is able to complement a *Δgcn4* mutant in *S. cerevisiae* [51]. When solely comparing the putative DNA binding domain of *cpcA*, the identity between CpcA and Gcn4 increases to 70% [51]. Therefore, the lack of homologous counterparts to GipA in other organisms does not necessarily mean that there is not a protein present in other fungi that functions similarly to GipA.

### High-Copy Expression of *gipA* Induces Gliotoxin Production

**Figure 4:**
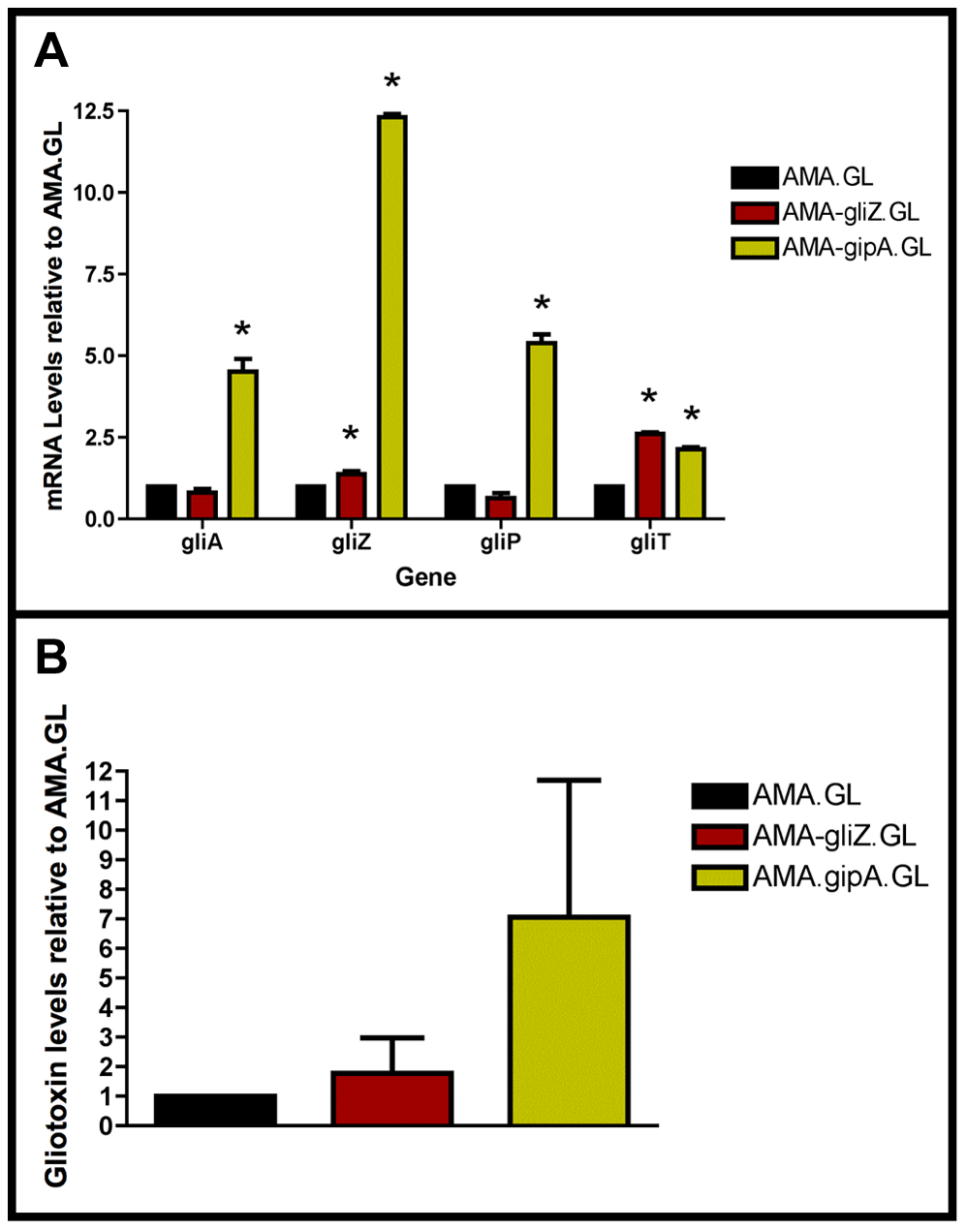
High-copy expression of *gipA* induces gliotoxin production. All cultures were grown in repressing conditions. Total RNA was isolated and quantified by dot blot analysis in triplicate. Gliotoxin levels were quantified by RP-HPLC using a standard curve and calculated as mg gliotoxin/g dry mycelial mass. All data sets are normalized to AMA.GL. (a) mRNA transcript levels of several gliotoxin cluster genes after 48 hrs of growth relative to AMA.GL. The results of one representative experiment of three independent experiments are shown as mean ± SD. (b) Gliotoxin levels in growth medium relative to AMA.GL. The data presented is an average of three biological replicates, shown as mean ± SD. The asterisk (*) indicates a statistically significant difference (p-value<0.05), compared to AMA.GL, calculated by one-way ANOVA and Tukey comparison test.

In a previous section, we showed that extra copies of *gipA* induce expression of *lacZ*, under the control of the *gliA* promoter, which suggests that GipA is inducing *gliA*. Since the gliotoxin biosynthesis cluster is coordinately regulated, extra copies of *gipA* should also induce other genes within the cluster. As expected, AMA-gipA.GL had a higher amount of *gliA* mRNA, than AMA.GL. Transcript levels of *gliA* in AMA-gipA.GL were 4.5-fold higher, compared to AMA.GL (Fig. 4a). The mRNA levels of the other gliotoxin-specific genes tested were also significantly higher in AMA-gipA.GL, compared to AMA.GL, as *gliZ* was induced 12-fold, *gliP* was induced 5-fold, and *gliT* was induced 2-fold (Fig. 4a). Gliotoxin production reflected what was seen with mRNA levels, as AMA-gipA.GL produced gliotoxin at a higher amount than AMA.GL (7-fold higher) (Fig. 4b). AMA-gliZ.GL was the positive control and showed the same pattern as AMA-gipA.GL, with respect to induction of the gliotoxin biosynthesis cluster.

### High-Copy Expression of *gipA* Possibly Affects Multiple Secondary Metabolite Clusters

To expand our view of GipA regulation, we performed a microarray analysis of AMA-gipA.GL vs. AMA.GL, grown in repressing conditions for 24 hrs. Of the 9,436 total genes analyzed, 443 genes were up-regulated >2-fold and 75 genes were down-regulated >2-fold in the AMA-gipA.GL strain (Fig. S1). There were several genes common to secondary metabolism clusters (e.g. transporters, oxidoreductases, methyltransferases, nonribosomal peptide synthetases and polyketide synthases) up-regulated (Table 1). Approximately 31 secondary metabolism clusters have been proposed using genomic mapping and microarray techniques [52], [53]. Of these 31 potential secondary metabolism clusters, 18 contained at least one gene that was up-regulated >2-fold in AMA-gipA.GL, compared to AMA.GL (Table 2 & Fig. S2). Based on microarray data done previously [52], loss of *laeA*, a global regulator of secondary metabolism, affected 13 of 22 identified secondary metabolite clusters. This suggests that GipA potentially induces numerous other secondary metabolism gene clusters in *A. fumigatus*, similar to LaeA. One caveat of this microarray, however, is that we measured the effects of high-copy expression of *gipA*, while the microarray done with LaeA compared a deletion strain to a wild-type and complemented strain. Therefore, based on these results, we cannot conclude that loss of *gipA* will have a significant effect on multiple secondary metabolite clusters, as is observed with loss of *laeA*.

**Table 1:** Genes commonly involved in secondary metabolism and possibly regulated by GipA.

Genes	Number up-regulated >2-fold
Transporters/Pumps	20
NRPS/PKS	9
Oxidoreductase	5
Methyltransferase	4
Acetyltransferase	1
C6 Transcription Factors	8

**Table 2:** 31 secondary metabolism clusters present in *A. fumigatus*.

Cluster	Cluster range*	Product
1	Afu1g17640–Afu1g17740	Unknown
2	Afu2g05730–Afu2g05840	Unknown
3	Afu2g17960–Afu2g18060	Ergot alkaloids: Festuclavine, Elymoclavine, Fumigaclavines A, B, and C [70]
4	Afu3g01290–Afu3g01600	Unknown
5	Afu3g02520–Afu3g02720	Unknown
6	Afu3g03350–Afu3g03470	Fumarylalanine & Triacetylfusarinine C [71]
7	Afu3g14560–Afu3g14760	Unknown
8	Afu3g15200–Afu3g15340	Unknown
9	Afu4g14380–Afu4g14850	Unknown
10	Afu5g00110–Afu5g00160	Unknown
11	Afu5g09940–Afu5g10220	Unknown
12	Afu6g03290–Afu6g03490	Unknown
13	Afu6g08540–Afu6g08560	Unknown
14	Afu6g09590–Afu6g09740	Gliotoxin [52]
15	Afu6g12040–Afu6g12110	Fumiquinazolines & Fumigaclavine C^1^ [72]–[74]
16	Afu7g00120–Afu7g00180	Neosartoricin [75]
17	Afu8g00170–Afu8g0250	Fumitremorgen B [52]
18	Afu8g02350–Afu8g02460	Unknown
19	Afu1g00980–Afu1g01010	Unknown
20	Afu1g10360–Afu1g10390	Fumigaclavine C [74]
21	Afu1g17120–Afu1g17240	Ferricrocin [71]
22	Afu2g01170–Afu2g01400	Unknown
23	Afu2g17510–Afu2g17600	Melanin [76]
24	Afu3g12890–Afu3g12960	Hexadehydroastechrome [77]
25	Afu3g13580–Afu3g13750	Unknown
26	Afu4g00210–Afu4g00230	Endocrocin [78], [79]
27	Afu5g00340–Afu5g00400	Unknown
28	Afu5g12700–Afu5g12740	Unknown
29	Afu6g13920–Afu6g14000	Unknown
30	Afu8g00370–Afu8g00520	Fumagillin [80]
31	Afu8g00530–Afu8g00580	Pseurotin A [81], [82]

The first 18 secondary metabolism clusters are the ones we predict to be positively regulated by GipA, while the last 13 did not have any genes up-regulated >2-fold from the microarray data.

*Cluster ranges for unknown products are defined in [52] or were predicted using SMURF at http://jcvi.org/smurf/index.php.

1 Although several genes in this cluster have been shown to be involved in production of fumiquinazolines, PesL (Afu6g12050) was recently revealed to be necessary for fumigaclavine C production.

### Deletion of *gipA* Significantly Reduces Gliotoxin Production

**Figure 5:**
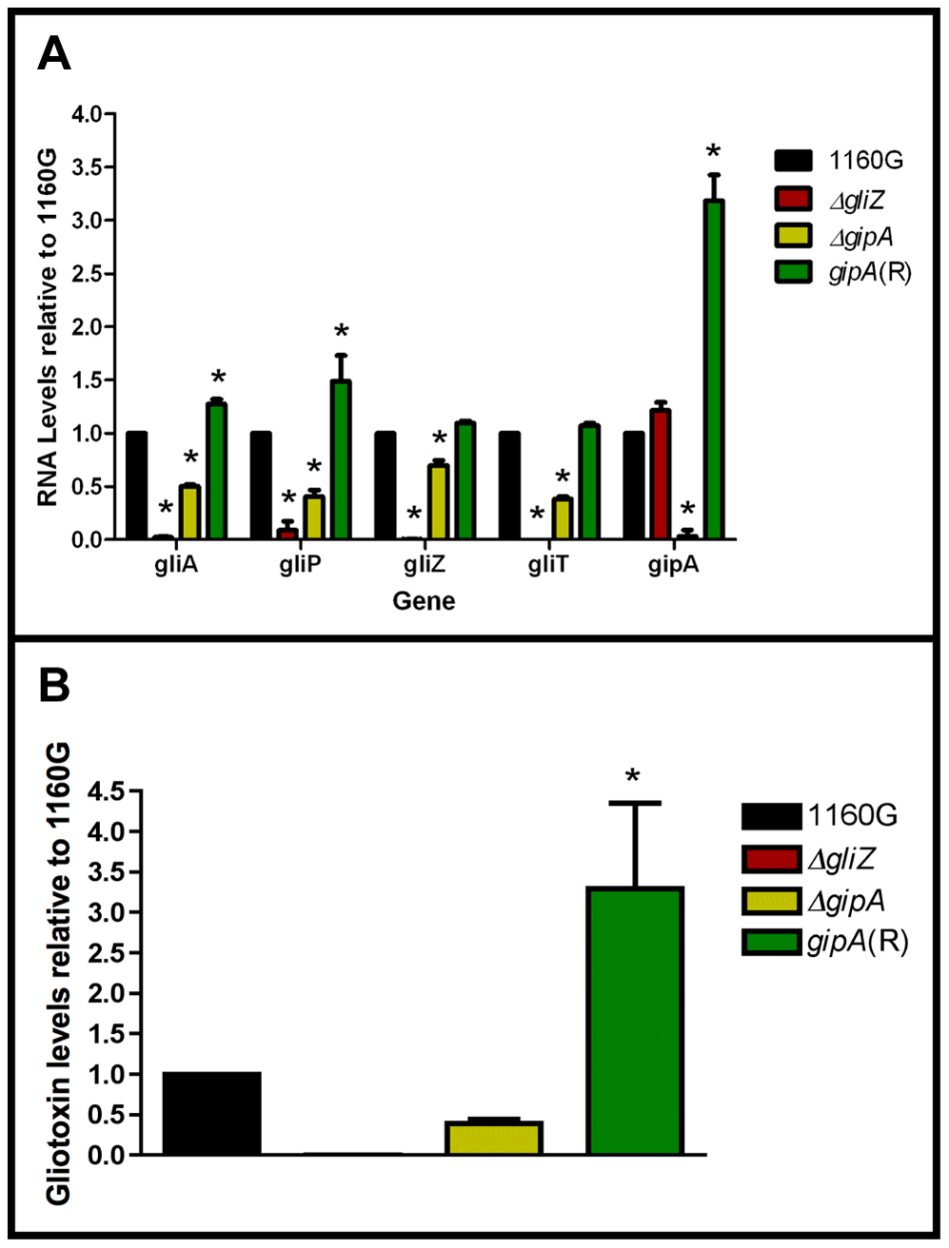
Loss of *gipA* negatively affects gliotoxin production. All cultures were grown for 48-repressing conditions. Total RNA was isolated and quantified by dot blot analysis in triplicate. Gliotoxin levels were quantified by RP-HPLC using a standard curve and calculated as mg gliotoxin/g dry mycelial mass. All data sets are normalized to 1160G. (a) mRNA transcript levels of several gliotoxin cluster genes relative to 1160G. The results of one representative experiment of three independent experiments are shown as mean ± SD. (b) Gliotoxin levels in growth medium relative to 1160G. The data presented is an average of three biological replicates, shown as mean ± SD. The asterisk (*) indicates a statistically significant difference (p-value<0.05), compared to 1160G, calculated by one-way ANOVA and Tukey comparison test.

Since GipA can induce gliotoxin production, we sought to discover if loss of *gipA* has any effect on the gliotoxin cluster. We replaced the coding region of *gipA* with *pyrG* and designated this strain as *ΔgipA*. We created a complemented strain, *gipA*(R), using a hygromycin resistance cassette (*hygroR*) as the selective marker. We also created a *gliZ* deletion strain as a control, since previous studies have shown that loss of *gliZ* results in a significant decrease in mRNA levels of gliotoxin-specific genes [12]. Loss of *gipA* caused a significant decrease in mRNA levels of *gliA, gliZ, gliP, and gliT* in non-repressing conditions, as most genes exhibited close to a 50% reduction (Fig. 5a). The gliotoxin biosynthesis cluster is not completely dependent on *gipA*, as there was still mRNA being made for the genes we tested. The *gipA* deletion mutant also produced significantly less gliotoxin than the 1160G control strain (50% reduction) (Fig. 5b). Gliotoxin-specific gene expression and gliotoxin production of *gipA*(R) were restored beyond wild-type levels, which demonstrates that the effect we observed with the *gipA* deletion was due to the absence of *gipA*. As expected, loss of *gliZ* caused almost a complete loss in gene expression for *gliA*, *gliP*, and *gliT* and abolished gliotoxin production. A *gliZ/gipA* double deletion mutant revealed a pattern of gene expression and gliotoxin production similar to the *gliZ* single deletion mutant (data not shown).

### Putative GipA Binding Sites Are Present Throughout the Gliotoxin Cluster

**Figure 6:**
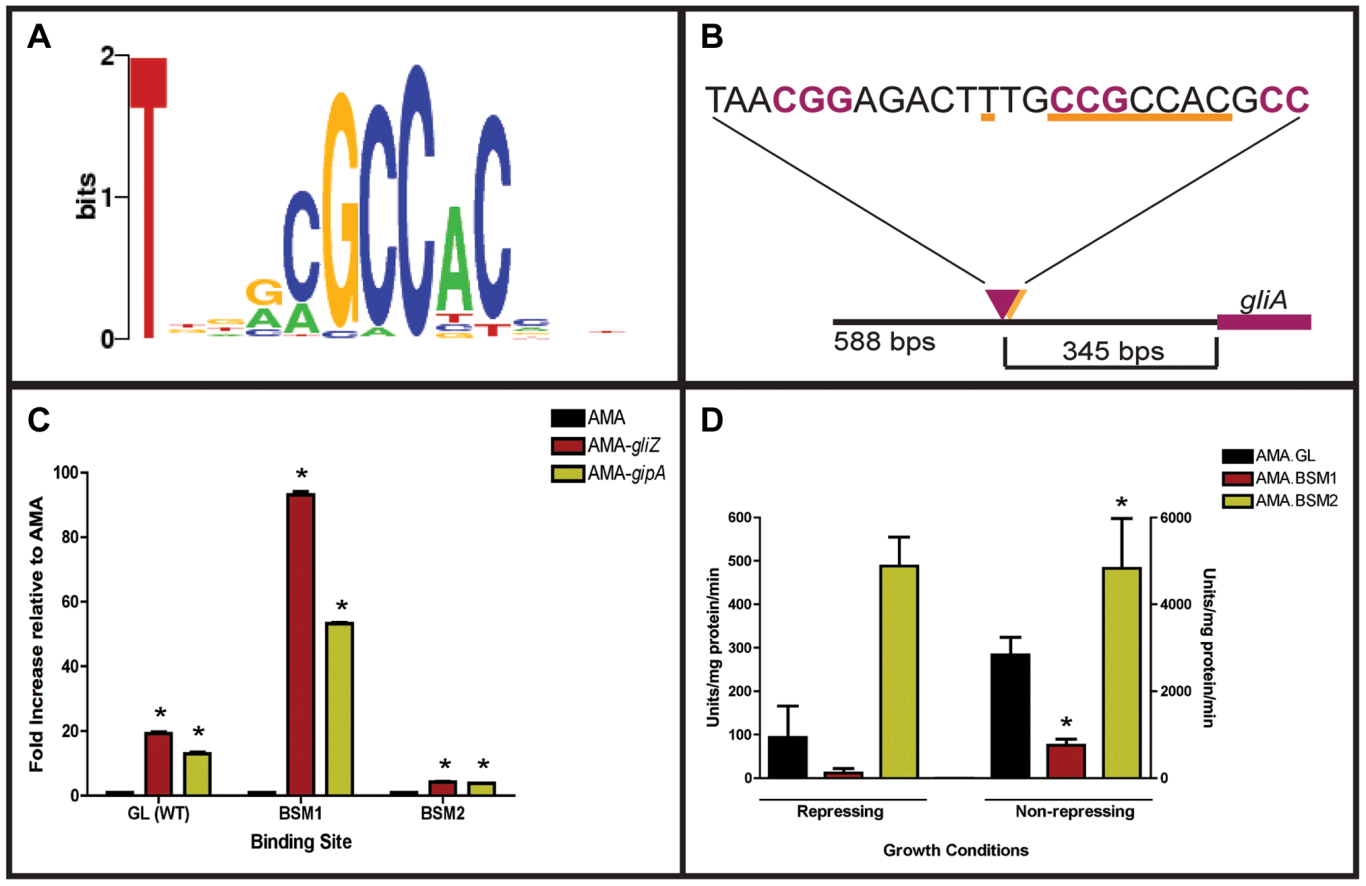
Characterization of the putative GipA DNA binding site. (a) Consensus sequence representing the putative DNA binding site for GipA obtained by protein binding microarray analysis. (b) Layout of putative GipA and GliZ binding sites in the *gliA* promoter region, relative to the *gliA* start site. Putative GliZ tandem repeats are purple and bolded and the putative GipA DNA binding site is underlined in orange. (c) Fold increase of LacZ levels of each strain in repressing conditions, relative to the AMA empty vector control. The results of one representative experiment of six independent experiments are shown as mean ± SD. (d) β-galactosidase activity of AMA.GL, AMA.BSM1, and AMA.BSM2 in both repressing and non-repressing conditions. The results of one representative experiment of three independent experiments are shown as mean ± SD. The asterisk (*) indicates a statistically significant difference (p-value<0.05) for each data set, compared to the AMA control strain, calculated by one-way ANOVA and Tukey comparison test.

Since GipA is a C_2_H_2_ transcription factor, it is likely that GipA is directly binding to DNA. We sought to identify a consensus sequence and to discover if this sequence was present within the gliotoxin biosynthesis cluster. A protein-binding microarray analysis identified a consensus DNA binding sequence for GipA (5′-TNNVMGCCNC-3′) (Fig. 6a). This putative sequence is 10 nucleotides, which coincides with one complete turn of the DNA double helix. Interestingly, there are 7 high-content positions (5′-VMGCCNC-3′), which would correlate with two C_2_H_2_ zinc finger DNA binding domains [54], and then a “T” residue two base pairs upstream of the 7 high content core positions. The protein-binding microarray verified direct DNA binding of GipA, as the purified DNA-binding domain, and not whole cell extract, was analyzed in the microarray. We analyzed the genomic sequence of the gliotoxin biosynthesis cluster to locate any potential GipA binding sites. Indeed, we found variations of this consensus sequence scattered throughout the gliotoxin biosynthesis cluster. In fact, we identified a possible GipA binding site within the intergenic region of *gliA* (5′-TTGCCGCCAC-3′ 315 bp upstream of the start site), as well as all other gliotoxin-specific genes, except *gliM* (Fig. 6b). Not all putative GipA DNA binding sites throughout the intergenic regions in the gliotoxin biosynthetic cluster contain the “T” residue in the 1 position, which means that either these sites are possibly only weakly recognized by GipA, if at all, or the “T” residue is not part of the actual GipA DNA recognition element.

### Mutational Analysis of the *gliA* Promoter Verifies the Presence of an Active GipA Binding Site

We created two mutant backgrounds: BSM1 (TTGCCGCCAC**C**TGCCGCCAC) and BSM2 (TTGCCGCCAC→TTG**GGTGAG**C), by mutating the putative GipA DNA binding site on the *gliA-lacZ* construct we used in the original screen. We measured *lacZ* expression with β-galactosidase assays in the presence of different high-copy plasmids (strains used in this study are listed in Table S2). When normalized to AMA.GL, AMA-*gipA*.GL displayed increased LacZ levels (13-fold), as was to be expected from previous results (Fig. 6c). High-copy expression of *gipA* in the BSM1 background also induced *lacZ* significantly (53-fold), relative to AMA.BSM1 (Fig. 6c). The magnitude of induction was enhanced with the BSM1 mutation when compared to the wild-type binding site (13-fold vs. 53-fold, respectively). This enhanced expression was not due to higher overall *lacZ* levels, as shown when comparing empty vector controls in all three backgrounds (Fig. 6d). AMA-*gipA*.BSM2 only weakly induced *lacZ* (4-fold), compared to AMA.BSM2 (Fig. 6c). This suggests that mutation of a core sequence in the putative GipA DNA binding site significantly reduces the ability of GipA to induce *lacZ*. Mutation of the 5′ “T” residue did not reduce GipA-specific *gliA* induction, but rather increased the response of *gliA* to GipA high-copy expression.

Interestingly, expression of *lacZ* in the pDONR AMA-*gliZ* strains followed a similar pattern to that of pDONR AMA-*gipA* strains (Fig. 6c). Therefore, *lacZ* was induced in AMA-*gliZ*.GL (19-fold) and AMA-*gliZ*.BSM1 (93-fold), compared to AMA.GL and AMA.BSM1, respectively. The level of induction was enhanced by the BSM1 binding site, compared to the wild-type binding site (19-fold vs. 93-fold, respectively). Furthermore, *lacZ* was only weakly induced in AMA-*gliZ*.BSM2, relative to AMA.BSM2 (4-fold). Therefore, mutation of the putative GipA DNA binding site is affecting the ability of GliZ to induce *gliA*.

### GipA Requires GliZ for Induction of Both *gliA* and *gliP*

**Figure 7:**
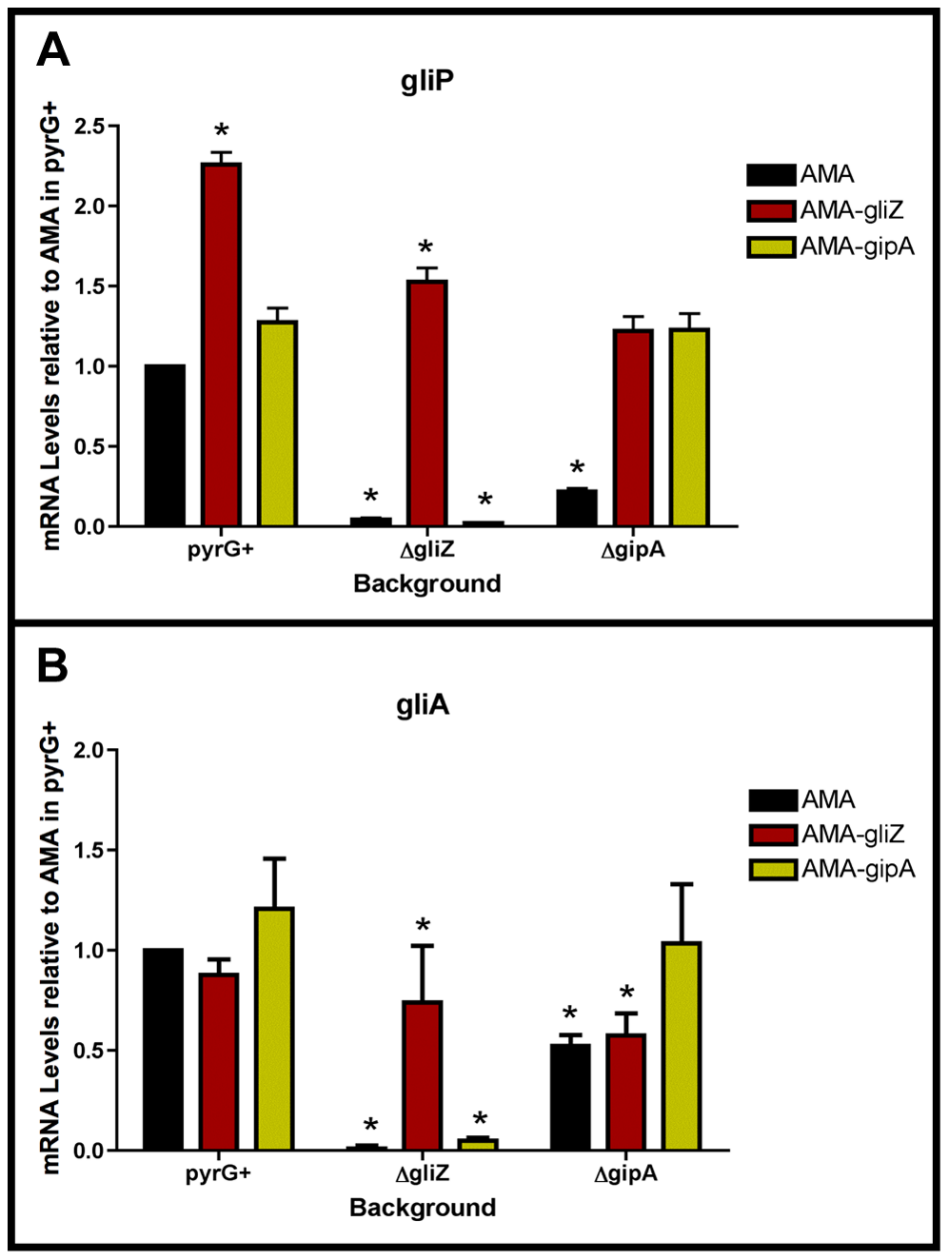
GliZ and GipA are dependent on each other for *gliA* induction. Cultures were grown in non-repressing conditions and mRNA was quantified by dot blot analysis in triplicate. Data are normalized to AMA.G (pyrG+ background). These graphs are an average of three biological replicates, shown as mean ± SD. (a) mRNA levels of *gliP* in all backgrounds relative to AMA.G. (b) mRNA levels of *gliA* in all backgrounds relative to AMA.G. The asterisk (*) indicates a statistically significant difference (p-value<0.05), compared to AMA.G, calculated by one-way ANOVA and Tukey comparison test.

To determine if GipA is dependent on GliZ for expression of *gliA*, we created a series of mutants and measured mRNA levels of *gliA* and *gliP*. High-copy expression plasmids used in previous sections were transformed into a wild-type background (AMA.G, AMA-*gliZ*.G, and AMA-*gipA*.G) and a *gliZ* deletion background (AMA.Z, AMA-*gliZ*.Z, and AMA-*gipA*.Z) (strains used in this study are listed in Table S2). All strains were grown in non-repressing conditions to induce expression of the gliotoxin biosynthesis cluster. In the pyrG+ background, AMA-gliZ.G and AMA-gipA.G had increased mRNA levels for *gliP*, while only AMA-gipA.G had increased mRNA levels for *gliA*, compared to AMA.G (Fig. 7). These changes were not significant because growth in non-repressing conditions already induced these genes to high levels, so having extra copies of GliZ or GipA did not greatly contribute to gene expression. In AMA.Z, mRNA for both *gliA* and *gliP* was almost completely undetectable, as to be expected from previous experiments. High-copy expression of *gliZ* brought transcript levels of both genes back to AMA.G levels (Fig. 7). AMA-gipA.Z displayed a reduction in mRNA levels similar to AMA.Z. For *gliA*, the level of mRNA present in AMA-gipA.Z was slightly higher (close to 5-fold) than what was observed for AMA.Z. However, the level of *gliP* mRNA did not exceed that of the AMA.Z empty vector control. Therefore, GipA was not able to induce *gliA* or *gliP* in the absence of GliZ. This suggests that GliZ is required for GipA to induce both *gliP* and *gliA*.

### GliZ Requires GipA for Induction of *gliA*, but not *gliP*

To determine if GliZ is dependent on GipA for expression of *gliA*, we created a series of mutants and measured mRNA levels of *gliA* and *gliP*. High-copy expression plasmids used in previous sections were transformed into a wild-type background (AMA.G, AMA-*gliZ*.G, and AMA-*gipA*.G) and a *gipA* deletion background (AMA.A, AMA-*gliZ*.A, and AMA-*gipA*.A) (strains used in this study are listed in Table S2). All strains were grown in non-repressing conditions to induce expression of the gliotoxin biosynthesis cluster. As expected from previous experiments, mRNA levels in AMA.A of both *gliA* and *gliP* were reduced significantly, 50% and 80%, respectively. Levels of *gliP* mRNA in AMA-gliZ.A were comparable to those of AMA.G; however mRNA levels of *gliA* were not significantly higher than background levels (AMA.A) (Fig. 7). This indicates that GliZ is not dependent on GipA for induction of *gliP*, however induction of *gliA* by GliZ does appear to be dependent on GipA.

## Discussion

**Figure 8:**
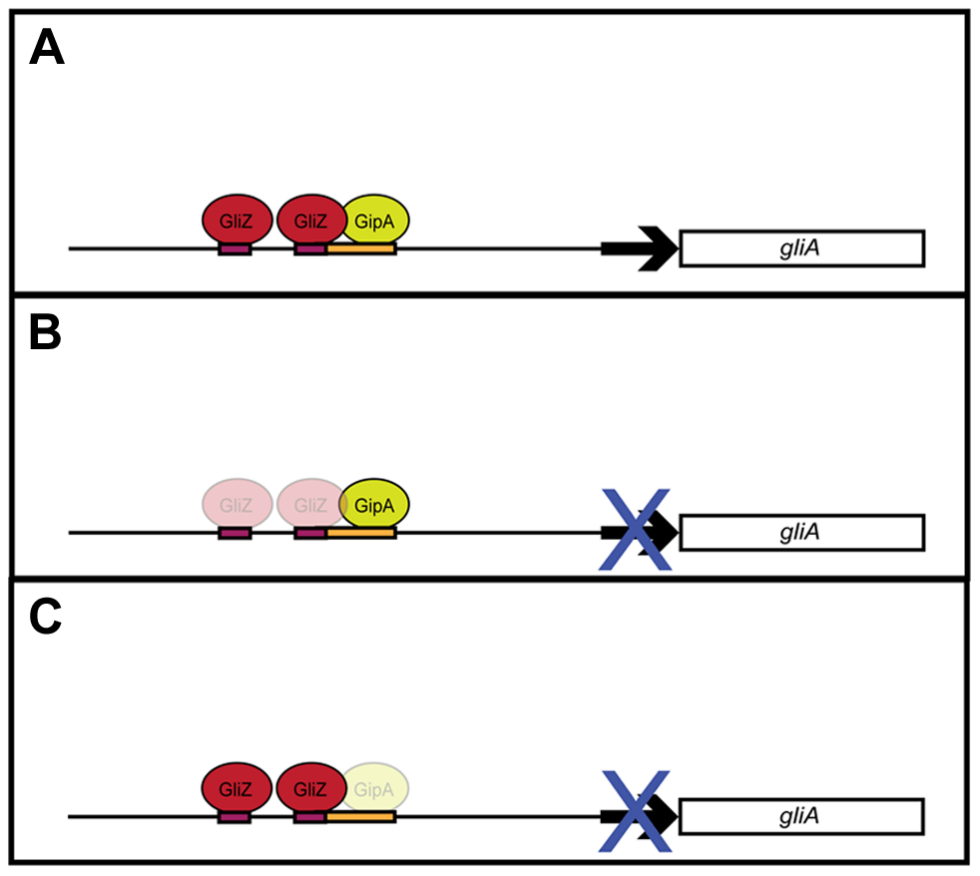
Model for *gliA* regulation involving GliZ and GipA. (a) We propose that GliZ and GipA both work interdependently to induce *gliA*. (b) With respect to the GliZ-GipA interdependent model, GipA cannot induce *gliA* in the absence of GliZ. (c) Furthermore, GliZ cannot induce *gliA* beyond a certain level in the absence of GipA. From our data, GipA is not essential to *gliA* expression, as there is still a moderate level of *gliA* mRNA produced when the gliotoxin cluster is induced, however, in the absence of GipA, high-copy expression of *gliZ* does not increase *gliA* mRNA levels beyond background levels. Our model is not meant to suggest that *gliA* is not induced at all in the absence of GipA, but merely that *gliA* induction specific to this GliZ-GipA interaction is lost if either protein is absent.

Although recent studies have revealed gliotoxin intermediates, which have led to a better understanding of the biosynthesis of gliotoxin, information on regulation of the genes involved in the biosynthesis pathway is lacking [28]. With the use of a high-copy inducer screen, our lab has uncovered a novel protein, GipA, which appears to be involved in the regulation of the gliotoxin cluster. GipA is a C_2_H_2_ transcription factor, which harbors an unusually long 5′ UTR. Furthermore, there are three μORFs within the 5′ UTR, which suggests that *gipA* may be under post-transcriptional control. There are two canonical C_2_H_2_ zinc finger DNA binding regions at the 3′ end of *gipA*, the first being X_2_-C-X_2_-C-X_12_-H-X_3_-H and the second being X_2_-C-X_2_-C-X_12_-H-X_5_-C, which is a natural variant [48], [49]. Two C_2_H_2_ zinc finger binding domains is consistent with a 6–8 base pair DNA recognition element [54]. The putative DNA binding site that we identified for GipA contains 7 high content positions (5′-TNNVMGCCNC-3′), in addition to a 5′ “T” residue two base pairs upstream of the core residues. Based on mutagenesis of the putative GipA DNA binding site in the *gliA* promoter, the 7 high content positions are likely part of an active GipA DNA binding site. While the “T” residue is not necessary for GipA-specific *gliA* induction, mutation of the “T” residue does enhance GipA-specific *gliA* expression. We have devised a possible model for the specific regulation of *gliA* that involves GliZ and GipA (Fig. 8). We propose that GliZ and GipA work together at the same binding site or in close proximity to induce *gliA*. In this model, GipA and GliZ are dependent on each other for inducing gene expression of *gliA*.

GipA appears to play an important role in gliotoxin biosynthesis, as high-copy expression of *gipA* causes increased gliotoxin production and loss of *gipA* causes a significant reduction in gliotoxin levels. Furthermore, GipA is possibly regulating other secondary metabolite clusters within *A. fumigatus*, similarly to LaeA, a global regulator of secondary metabolism [52]. In contrast to LaeA, though, GipA is a C_2_H_2_ transcription factor, which likely directly binds to DNA recognition sites. Other C_2_H_2_ transcription factors, such as PacC, NsdC, NsdD and CreA, have also been identified as regulators of secondary metabolism. PacC regulates a wide range of genes in response to ambient pH, including those involved in secondary metabolism. Penicillin production in *A. nidulans* is induced in alkaline conditions, while sterigmatocystin production is repressed [4], [55], [56]. NsdC and NsdD are both involved in sexual development in *A. nidulans*. However, recent work by Jeffrey Cary et al. [57] supported additional roles for NsdC and NsdD in asexual development and secondary metabolism in *A. flavus*. Although AflR expression was not affected, loss of either *nsdD* or *nsdC* resulted in decreased expression of other genes in the aflatoxin biosynthesis gene cluster [57]. Finally, CreA is a global regulator of carbon catabolite repression in fungi. In addition to carbon metabolism, CreA also affects secondary metabolism, as cephalosporin production is reduced in *Acremonium chrysogenum* in response to high glucose [56], [58]. It is possible that GipA serves to enhance expression of the gliotoxin cluster in certain environmental conditions. Both high-copy expression and loss of *gipA* affect *gliZ* expression, so the effects we see for the entire cluster could be the direct consequence of GipA binding to each promoter region, or an indirect consequence of GipA partially regulating *gliZ*, which in turn regulates other genes within the cluster. Loss of both *gliZ* and *gipA* does not give conclusive evidence for either possibility, as loss of *gliZ* alone completely abolishes gliotoxin production. None of the mutants tested displayed abnormal growth rates or attenuated virulence in a *Drosophila melanogaster* model system, indicating that high-copy expression of *gipA* or loss of *gipA* does not affect overall fitness of *A. fumigatus* (data not shown).

Our data show a dependency between GipA and GliZ with respect to *gliA* expression. Firstly, there is a putative GliZ DNA binding site embedded within a GipA DNA binding site in the *gliA* 5′ UTR. Although a GliZ binding site has not yet been experimentally determined, one has been predicted (TCGGN_3_CCGA). This sequence is present in the intergenic region of every gene within the gliotoxin cluster, except *gliZ* and *gliA* [14]. Recognition of these sequences by Zn_2_Cys_6_ binuclear finger transcription factors is often very specific and even a slight change to the length or base composition of the linker sequence can result in reduced binding *in vivo* [15], [17], [18]. Within the *gliA* promoter region, a sequence overlapping the GipA binding site has the CGG-CCG inverted repeats common to Zn_2_Cys_6_ binuclear finger transcription factor binding sites, but the linker sequence is longer (8 bps) than that of the predicted GliZ DNA binding site (3 bps). Mutation of the CCG repeat almost completely abolishes *gliZ*-mediated induction of *gliA*. This suggests that either the prediction for the GliZ DNA binding site is incorrect and is actually CGGN_8_CCG or that the GliZ DNA binding site within the *gliA* UTR is unique. Due to the position of the putative GliZ DNA binding site, mutation of this CCG also changes the core sequence of the putative GipA DNA binding site. Accordingly, *gipA*-mediated induction of *gliA* is almost completely abolished.

Dependent dual regulation of two transcription factors has been uncovered in other organisms. For example, *in A. nidulans*, FlbB and FlbD both bind in close proximity to the *brlA* promoter to regulate asexual development through *brlA* activation [59], [60]. Furthermore, FlbB does not bind to the *brlA* promoter in the absence of FlbD, indicating that these two transcription factors are dependent on each other for DNA binding and activation of *brlA* [60]. Although the mutational analysis of the *gliA* promoter raises the possibility of a GliZ-GipA dependency, it does not give conclusive evidence. One could argue that disruption of the GliZ DNA binding site causes loss of *gliA* induction that indirectly abolishes *gipA*-mediated induction of *gliA*. However, our hypothesis of a dependency between GipA and GliZ with respect to *gliA* expression is further supported by the fact that GliZ cannot induce *gliA* in the absence of GipA. As expected, loss of *gliZ* severely decreases mRNA levels of *gliA*, which cannot be rescued by high-copy expression of *gipA*. Interestingly, loss of *gipA* also significantly decreases mRNA levels of *gliA*, which cannot be rescued by high-copy expression of *gliZ*. Clearly, these two proteins work together to induce *gliA* beyond basal levels in non-repressing conditions. This pattern of dependency is unique to *gliA* expression, as *gliP* is induced by high-copy expression of *gliZ*, even in the absence of *gipA*.

**Figure 9:**
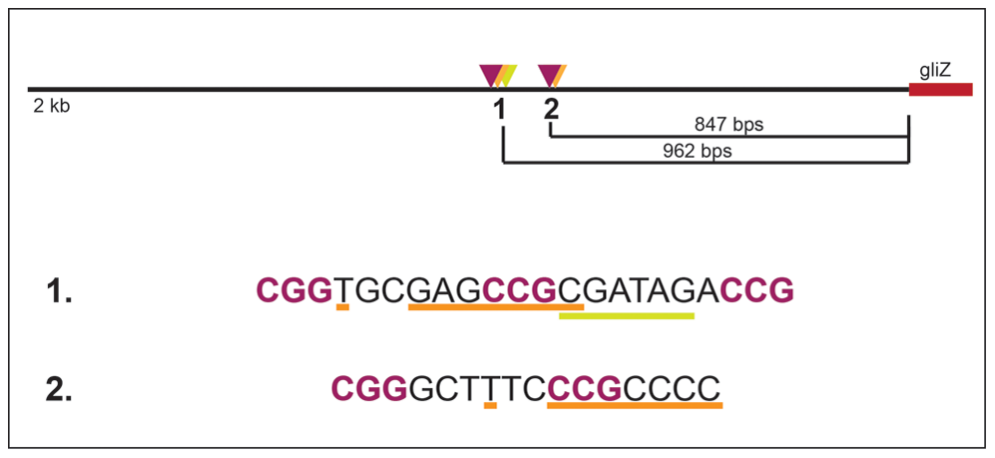
Layout of two potential GipA binding sites that are embedded in putative GliZ binding sites in the *gliZ* promoter. GliZ putative trinucleotide repeats are purple and bolded, the putative GipA binding sites are underlined in orange, and binding cluster 1 additionally contains an AreA recognition element (underlined in yellow).

Together, these data support a model in which GliZ and GipA are working together to regulate *gliA* (Fig. 8). There appears to be a dependency with regards to *gliA* expression that we demonstrated in our experiments. This model does not apply to every gene within the gliotoxin cluster, as no other genes have a GipA binding site embedded within a possible GliZ binding site, except *gliZ*, although these sequences are located farther upstream of the *gliZ* start site (Fig. 9). Possibly, GipA serves to aid GliZ in binding to the *gliA* promoter region, as this binding site is different from the others present in the gliotoxin cluster. Although there are possible GipA binding sites in all gliotoxin gene promoters, except *gliM*, we cannot say with certainty whether GipA is directly binding to these other promoter regions or if GipA is simply binding in the *gliA* promoter in conjunction with GliZ. This adds to mounting evidence that genes within a gene cluster are typically coordinately regulated, but can also be individually expressed in response to certain stimuli. It is possible that *gliT* and *gliA* are both independently regulated to protect the fungus from exogenous gliotoxin, although *gliA* was not induced in a *ΔgliZ* mutant in the presence of exogenous gliotoxin as *gliT* was [19]. Another possibility is that *gliA* is independently regulated to aid in the transport and/or expression of other secondary metabolism clusters in *A. fumigatus*. There has been evidence in other fungal species that crosstalk between these gene clusters exists [61].

## Materials and Methods

All primers used in this study are listed in **Table S1**.

### Strains and Growth Conditions

All strains used in this study and genotypes are listed in **Table S2**. We maintained Af293.1, Af293.1-GL, 1160, Af293.1-BSM1, and Af293.1-BSM2 on YAG medium supplemented with uridine and uracil (0.5% yeast extract, 1% glucose, trace elements and vitamin mix as modified [62], 10 mM MgCl_2,_ 1.5% agar, 5 mM uridine, and 10 mM uracil,). We grew AMA.G, AMA.Z, AMA.A, AMA-gliZ.G, AMA-gliZ.Z, AMA-gliZ.A, AMA-gipA.G, AMA-gipA.Z, and AMA-gipA.A on YAG medium with 400 µg/ml hygromycin. We maintained all other strains on YAG medium. Unless otherwise noted, we grew all strains at 37°C for 48 hrs. For phenotypic growth assays of high-copy and deletion strains, we inoculated approximately 1000 spores of each strain onto MMVAT (1× MM salts [20 mM ammonium tartrate, 7 mM KCl, 2 mM MgSO_4_·7H_2_O], 1% glucose, 12 mM KPO_4_ pH 6.8, trace elements, vitamin mix as modified [62], and 1.25% agar), MMVAT with 10 µg/ml gliotoxin, and YAG. MMVAT plates were incubated for 72 hrs. We repeated plate growth assays twice for a total of three independent tests. We measured radial growth of each colony and scanned plates on the final day of growth.

### High-Copy Inducer Screen

We used fusion PCR (f-PCR) to create a *gliA^P^*-*lacZ*-*gliA^T^* construct. For the first reaction, we created three cassettes: *gliA* 5′^P^, *lacZ*, and *gliA* 3′^T^, using primer pairs GliA F1 and GliA 5′ R, lacZ F and lacZ R, and GliA 3′ F and GliA R, respectively. We obtained these three fragments by PCR using e2TAK DNA polymerase (Takara Bio Inc., Otsu, Shiga, Japan, Otsu, Shiga, Japan) following manufacturer recommendations. The *gliA* 5′^P^ fragment had a 3′ extension identical to the first 15 base pairs of *lacZ*. The *lacZ* fragment had a 5′ extension identical to the last 15 base pairs of *gliA* 5′^P^ and a 3′ extension identical to the first 15 base pairs of *gliA* 3′^T^. The *gliA* 3′^T^ fragment had a 5′ extension identical to the last 15 base pairs of *lacZ*. We used Af293 genomic DNA as template for the *gliA* 5′^P^ and *gliA* 3′^T^ regions and λGT11 as template for *lacZ*.

For the second reaction, we fused the three fragments together using GliA F1 and GliA R as primers. We amplified a 50 µl reaction containing 50 fmol of each fragment, 0.3 µM of each primer, 500 µM of deoxynucleoside triphosphates, buffer 3 at a 1× concentration, and 1 µl of Expand Long DNA Template Mix (Roche Applied Science, Indianapolis, IN) per manufacturer's instructions (briefly, 94°C for 2 min, 10 cycles of 94°C for 10 sec, 62°C for 30 sec, 68°C for 4.5 min and 15 cycles of 94°C for 15 sec, 62°C for 30 sec, 68°C for 4.5 min, increasing the final extension time by 20 sec with each cycle).

We cloned the fusion product into pDONR HPH A [63] using a BP recombination reaction (Invitrogen, Grand Island, NY). We transformed the reaction mix into TOP10 cells (Invitrogen, Grand Island, NY) by electroporation, as recommended by the manufacturer. We grew the transformation mix on LB (1% tryptone, 0.5% yeast extract, 1× SOB salts [10 mM NaCl, 2.5 mM KCl], 1.5% agar) +50 µg/ml kanamycin at 37°C overnight. We picked colonies and transferred to 2 ml of LB liquid +50 µg/ml kanamycin to grow overnight in a 37°C shaking incubator. We isolated plasmid DNA from each culture using a miniprep kit (Qiagen, Hilden, Germany). We digested plasmid DNA with specific enzymes to verify the correct insertion. We designated this vector as pDHGL.

We grew Af293.1 in MAG medium supplemented with uridine and uracil (2% malt extract, 0.2% peptone, 1% glucose, trace elements and vitamin mix as modified [62], 2% agar, 5 mM uridine, 10 mM uracil). We performed the transformation as previously described [64], keeping pDHGL as a circular vector. We grew transformants on MMVAT supplemented with 5 mM uridine, 10 mM uracil, 0.2 M sucrose, and 400 µg/ml hygromycin at 37°C for 3–5 days. We identified the presence of pDHGL by Southern hybridization [65] of the *lacZ* coding region (Fig. S3). We also streaked transformants onto MMVSN supplemented with uridine and uracil (as described above for MMVAT, except 1×MM salts contain 20 mM sodium nitrate instead of ammonium tartrate) and 40 µg/ml X-gal to grow at 37°C for 2 days. We screened for transformants that grew in the presence of hygromycin and developed a blue pigment on X-gal, signaling that *lacZ* expression was working properly. We designated this strain as Af293.1-GL.

For the first round of the high-copy inducer screen, we grew Af293.1-GL in MAG supplemented with uridine and uracil. We transformed the AMA1-Not1 *A. fumigatus* genomic library [43] into Af293.1-GL as described previously [64], but with changes. We combined each transformation mix with 50 mls of CM top agar (MMVAT, as described above, 0.1% yeast extract, 0.2% peptone, 0.1% tryptone, 1% CM supplement [27 mM adenine HCl, 33.5 mM methionine, 173 mM arginine, and 1.3 mM riboflavin], and 1% agar) supplemented with 1 M sucrose and spread the mixture over 10 plates (5 ml/plate). We grew transformants on CM supplemented with 0.2 M sucrose and 40 µg/ml X-gal at 37°C for 3–5 days. We screened for transformants that were both prototrophic for uridine and uracil and producing a blue pigment. We prepared genomic DNA from transformants [66] and transformed 1 µl of genomic DNA into TOP10 cells (Invitrogen, Grand Island, NY) by electroporation, as recommended by the manufacturer. We grew the transformation mix on LB +100 µg/ml ampicillin at 37°C overnight. We picked colonies and transferred to 2 ml of LB liquid +100 µg/ml ampicillin to grow overnight in a 37°C shaking incubator. We isolated plasmid DNA from each culture using a miniprep kit (Qiagen, Hilden, Germany) and digested it with KpnI to identify individual plasmids.

For the second round of the high-copy inducer screen, we grew Af293.1-GL in MAG supplemented with uridine and uracil. We transformed each individual plasmid isolated from the first round of the genetic screen as described above, but with changes. We plated two amounts of protoplasts (20 µl and 100 µl) each in 4 mls of MMVAT top agar (as described above but with 1% agar) supplemented with 1 M sucrose. We grew transformants on MMVAT supplemented with 0.2 M sucrose and 40 µg/ml X-gal at 37°C for 3–4 days and put plates in the 4°C refrigerator to facilitate blue pigment production. Plasmids causing at least 80% of colonies to turn blue were sequenced using primers AMA-NotI F and AMA-NotI R. We also grew transformants in 10 mls CM at 37°C stationary overnight. We collected mycelia, froze in liquid nitrogen, and lyophilized overnight. We collected total protein and performed β-galactosidase assays to measure LacZ levels quantitatively (detailed below).

For the third round of the high-copy inducer screen, we PCR amplified individual genes from genomic library plasmids, flanked by native 5′ and 3′ non-coding regions. For *gipA* (Afu6g01910), we used the primer pair 6g01910 F and 6g01910 R. We amplified the fragments from Af293 genomic DNA using e2TAK DNA polymerase (Takara Bio Inc., Otsu, Shiga, Japan) following manufacturer recommendations. We cloned the PCR fragments into pDONR AMA [63] with a BP recombination reaction (Invitrogen, Grand Island, NY). We transformed the reaction mix into TOP10 cells (Invitrogen, Grand Island, NY) by electroporation, as recommended by the manufacturer. We grew the transformation mix on LB +100 µg/ml ampicillin at 37°C overnight. We picked colonies and transferred to 2 ml of LB liquid +100 µg/ml ampicillin to grow overnight in a 37°C shaking incubator. We isolated plasmid DNA from each culture using a miniprep kit (Qiagen, Hilden, Germany). We digested plasmid DNA with specific enzymes to verify the correct insertion. We designated the *gipA*-containing vector as pDONR AMA-*gipA*. We created a control vector, pDONR AMA-*gliZ*, which contained the *gliZ* coding region flanked by promoter and terminator regions. We generated this vector as described above for pDONR AMA-*gipA* using primers GliZ attB 1 and GliZ attB 2.

We grew Af293.1GL in MAG medium, supplemented with uridine and uracil. We performed the transformation as previously described [64], with changes, using pDONR AMA and pDONR AMA-gliZ as controls. After the 3 hour incubation of protoplasts, we carried out all reactions at half the specified volume. We used 500 ng–750 ng of circular vector DNA for each reaction. We plated two amounts of protoplasts (20 µl and 50 µl) each in 4 mls of MMVAT top agar supplemented with 1 M sucrose. We grew transformants on MMVAT medium supplemented with 0.2 M sucrose at 37°C for 2–3 days. To measure LacZ levels quantitatively, we grew transformants in 10 mls CM at 37°C stationary overnight. We collected mycelia, froze in liquid nitrogen, and lyophilized overnight. We collected total protein and performed β-galactosidase assays (detailed below). We chose three strains to use for future analysis: AMA.GL, AMA-gliZ.GL, and AMA-gipA.GL.

### λ Phage Library Screen

We isolated *gipA* cDNA clones from a λ phage library constructed with the UniZAP vector and poly (A) + mRNA, as described by the manufacturer (Stratagene, La Jolla, California). We performed a primary screen of the λ phage library as recommended by manufacturer (Stratagene, La Jolla, California). We used the *gipA* coding region as a probe [65]. We excised plugs containing positive plaques and placed them in SM (.1 M NaCl, 10 mM MgSO_4_·7H_2_O, 50 mM Tris·HCl [pH 7.5], and .01% gelatin) at 4°C overnight. After determining pfu concentration of plaques, we performed a secondary screen, as performed for the primary screen. We PCR amplified cDNA from excised phagemids using e2TAK DNA polymerase (Takara Bio Inc., Otsu, Shiga, Japan) and following manufacturer recommendations. We used M13 F and M13 R as primers for the reaction. Based on the PCR band sizes, we picked colonies from selected plates and transferred to 2 ml of LB liquid +100 µg/ml ampicillin to grow overnight in a 37°C shaking incubator. We isolated plasmid DNA from each culture using a miniprep kit (Qiagen, Hilden, Germany) and sequenced the cDNA insert using M13 F and M13 R primers. We used DNASTAR software (DNASTAR Inc.) to assemble and analyze the sequences obtained from the library screen.

### RNA Dot Blot Analysis

For all Dot Blot assays, we grew stationary cultures in 25 mls of CM (repressing) (described above) or CD (non-repressing) (87.6 mM sucrose, 35.3 mM sodium nitrate, 5.8 mM K_2_HPO_4_, 2 mM MgSO_4_·7H_2_O, 6.7 mM KCl, and 0.025 mM ferrous ammonium sulfate) at a concentration of 5×10^6^ spores/ml at 37°C for 48 hrs. We included 400 µg/ml hygromycin in dot blot assays involving AMA.G, AMA.Z, AMA.A, AMA-gliZ.G, AMA-gliZ.Z, AMA-gliZ.A, AMA-gipA.G, AMA-gipA.Z, and AMA-gipA.A. We prepared total RNA from freeze-dried mycelia using the TRIzol method [62]. We incubated total RNA with denaturing solution (50% formamide, 16%formaldehyde, 1× borate buffer [20× borate buffer: 0.4 M Boric Acid, 4 mM EDTA, pH 8.3 with NaOH], 0.025% bromophenol blue) for 10 min at 65°C. We quenched samples on ice for 10 min, then added equal volume 20× SSC (3 M NaCl, 0.3 M sodium citrate, pH 7.0). We placed a nylon membrane that had been equilibrated in 10× SSC for 10 min into a 96 well dot blot apparatus attached to a vacuum manifold. We collected samples, each containing 3 µg of RNA unless otherwise noted, in 100 µl volumes by aspiration. We aspirated 50 µl of 10× SSC through the membrane in duplicate immediately before and after samples were collected. Once all samples were aspirated, we air dried nylon membranes overnight and then baked them in an oven for 2 hrs. For prehybridization and hybridization, we sealed nylon membranes in a bag using a heated sealer. We prehybridized membranes for 4–6 hrs at 42°C and hybridized membranes overnight at 42°C. For DNA probes, we only used the coding region of each gene of interest (*gliA*, *gliP*, *gliZ*, *gliT*, *gipA*, and actin). After hybridization, we washed membranes as we would for Southern hybridization [65] and exposed them to a Typhoon 8600 PhosphorImager (GE Healthcare Life Sciences, Pittsburgh, PA) overnight. We quantified the intensity of hybridization using ImageQuant5.1 software (GE Healthcare Life Sciences, Pittsburgh, PA).

### Gliotoxin Extraction and HPLC Analysis

We extracted spent culture medium from RNA dot blot assays in 15 ml chloroform for 30 min at room temperature on an orbital shaker set to 250 rpm. We transferred the chloroform phase to a 50 ml conical tube (BD Biosciences, San Jose, California) and repeated the extraction twice for a total of 45 mls of chloroform. We dried open tubes under a hood until the chloroform was completely evaporated. We added 15 mls of chloroform to each tube and mixed, to concentrate the extracted material at the bottom. We dried open tubes under a hood until chloroform was completely evaporated. We added 1 ml of methanol to each tube and mixed to dissolve all methanol-soluble substances and transferred extracts to microcentrifuge tubes (Fisher Scientific, Pittsburgh, PA). We dried open tubes under a hood until all methanol was completely evaporated.

We dissolved extracts in 50 µl of dimethyl sulfoxide (DMSO), spun tubes to pellet insoluble debris, and transferred DMSO to fresh microcentrifuge tubes. We quantified gliotoxin levels by running samples through a reverse-phase high performance liquid chromatography (RP-HPLC) system with a Waters 996 photodiode array detector (Waters, Milford, MA). We ran samples through a Sonoma 2.1×250 mm C18 column (100 Å pore size) packed with 5 µM particles (VWR, Radnor, PA). The mobile phase consisted of H_2_O, 0.1% TFA (solution A) and 100% Acetonitrile, 0.1% TFA (solution B): 10% B up to 80% B over 30 min. The injection volume was 10 µl and flow rate was set at 0.4 ml/min. Gliotoxin eluted from the column at 14.7 min and absorbance was read at 268 nm wavelength. We determined gliotoxin concentrations by interpolation from a 9 point standard curve (39 ng to 10 µg) prepared using purified gliotoxin (Sigma-Aldrich Corp., St. Louis, MO).

### Microarray

The DNA amplicon microarray for Af293 was created previously [67]. We grew AMA.GL and AMA-gipA.GL in 25 mls CM (described above) at 37°C for 24 hrs in stationary cultures. The spore concentration was 5×10^6^ spores/ml. We used two independent biological replicates for AMA.GL and AMA-gipA.GL growth assays. We prepared total RNA from freeze-dried mycelia using the TRIzol method [62]. We carried out RNA labeling reactions and hybridizations as described in the J. Craig Venter Institute Microarray Protocols (http://pfgrc.jcvi.org/index.php/microarray/protocols.html). We repeated all the hybridizations in dye-swap sets. We scanned and analyzed hybridized slides as described previously [67]. We averaged all replicates for the official data used in our analysis.

### Virulence Assays with Toll-Deficient *D. melanogaster*

We injected the dorsal side of the thorax of CO_2_-anesthetized, adult, Toll-deficient *D. melanogaster* flies with a sterile 0.25 mm needle that had been dipped in a solution containing 10^7^ spores/ml of *A. fumigatus* conidia. We infected 20–25 flies per strain for each virulence assay, which was repeated at least twice. Flies were kept in a 29°C incubator to maximize susceptibility to microbial challenge and monitored for 7 days. Flies that died within 3 hrs of the injection were not included in the survival graph, as these flies most likely died as a result of the puncture wound.

### Deletion and Complementation of *gipA* in Af1160

We amplified the 5′ flanking region (FR) and the 3′ FR from *gipA* using primers 01910 5′ F, 01910 5′ R, 01910 3′ F, and 01910 3′ R. We engineered a unique NotI site into 01910 5′ F to linearize the final deletion construct. We amplified the fragments from Af293 genomic DNA using e2TAK DNA polymerase (Takara Bio Inc., Otsu, Shiga, Japan) and following manufacturer recommendations. We cloned the *gipA* 5′ FR into pDONR P4-P1R and we cloned the *gipA* 3′ FR into pDONR P2R-P3, using BP recombination reactions (Invitrogen, Grand Island, NY). We transformed BP reaction mixes into TOP10 cells (Invitrogen, Grand Island, NY) by electroporation, as recommended by the manufacturer. We grew transformed cells on LB (described above) +50 µg/ml kanamycin at 37°C overnight. We picked colonies and transferred to 2 ml LB liquid +50 µg/ml kanamycin to grow overnight in a 37°C shaking incubator. We isolated plasmid DNA from each culture using a miniprep kit (Qiagen, Hilden, Germany). We digested plasmid DNA with specific enzymes to verify the correct fragment orientation.

To create the deletion constructs, we combined pDONR P4-P1R-*gipA* 5′ FR, pDONR P2R-P3-*gipA* 3′ FR, and pDONR 221-*AnpyrG* [63] in an LR recombination reaction with pDEST R4-R3 as the destination vector (Invitrogen, Grand Island, NY) [68]. We transformed the LR reaction mix into TOP10 cells (Invitrogen, Grand Island, NY) by electroporation, as recommended by the manufacturer. We grew transformed cells on LB +100 µg/ml ampicillin at 37°C overnight. We picked colonies and transferred to 2 ml LB liquid +100 µg/ml ampicillin to grow overnight in a 37°C shaking incubator. We isolated plasmid DNA from each culture using a miniprep kit (Qiagen, Hilden, Germany). We digested plasmid DNA with specific enzymes to verify the correct fragment orientation. We grew bacterial cultures containing the correct plasmid in 250 ml LB liquid +100 µg/ml ampicillin overnight in a 37°C shaking incubator. We purified plasmid DNA by banding on cesium chloride ethidium bromide gradients [65]. We designated the plasmid pDEST R4-R3-*gipA* 5′ FR-*AnpyrG*-*gipA* 3′ FR.

We grew *A. fumigatus* 1160 (obtained from FGSC) in MAG supplemented with uridine and uracil. We performed the transformation as previously described [64], linearizing the deletion construct with NotI to facilitate homologous recombination. We grew transformants on MMV supplemented with 0.2 M sucrose at 37°C for 3–5 days. We screened for mutants that were prototrophic for uridine and uracil. We prepared genomic DNA from transformant strains [66], and we identified deletion mutants by Southern blot analysis (Fig. S4) [65]. We made a DNA probe using the *gipA* 5′ and 3′ FRs to verify *ΔgipA*.

To create a vector for complementation, we PCR amplified the *gipA* coding region, flanked by a 3 kilobase promoter region and 500 base pair terminator region, using primers gipA 3 kb F and 6g01910 R. We amplified the fragment from Af293 genomic DNA using e2TAK DNA polymerase (Takara Bio Inc., Otsu, Shiga, Japan) and following manufacturer recommendations. We cloned the PCR product into pDONR HPH B [63], as described previously for pDHGL and designated this vector pDONR HPH-*gipA*. We grew *ΔgipA* in MAG medium and performed a transformation as previously described for pDHGL. For Southern hybridization, we made a DNA probe using the coding region of *gipA* (Fig. S5). We designated this strain as *gipA*(R).

We obtained controls (1160G and *ΔgliZ*) for growth assays. We created 1160G by transforming pDONR G [63] into Af1160 and we created *ΔgliZ* by transforming pDEST R4-R3-*gliZ* 5′ FR-*AnpyrG*-*gliZ* 3′ FR into Af1160, performed as described above for *ΔgipA*. We created the *gliZ* deletion construct as described previously for *ΔgipA* using primers gliZ 5′ F, gliZ 5′ R, gliZ 3′ F, and gliZ 3′ R. We used *gliZ* 5′ and 3′ flanking regions as a DNA probe for Southern hybridization (Fig. S4).

### Construction of *ΔgliZ/ΔgipA*

We grew *ΔgipA* on YAG supplemented with uridine and uracil +1 mg/ml 5-Fluororotic acid (5-FOA) at 37°C. Colonies that reverted to a *pyrG*- phenotype grew as outgrowths from the original streak. We prepared genomic DNA from these *pyrG*- outgrowths [66] and tested them for the presence of *gipA* by Southern hybridization (Fig. S6) [65]. We used the *gipA* coding region as a DNA probe. We designated this mutant as *ΔgipA.0.* We transformed pDEST R4-R3-*gliZ* 5′ FR-*AnpyrG*-*gliZ* 3′ FR into *ΔgipA.0*, as described above for *ΔgipA*. We used *gliZ* 5′ and 3′ flanking regions as a DNA probe for Southern hybridization (Fig. S7).

### Protein Binding Microarray

We amplified the C_2_H_2_ DNA binding region from *gipA* using primers gipA C2H2 F and gipA C2H2 R. We used Af293 as template and AccuPrime Pfx DNA polymerase (Life Technologies, Grand Island, NY) and per manufacturer's recommendations. We cloned the PCR product into pDONR 221 using a BP recombination reaction (Invitrogen, Grand Island, NY). We transformed the reaction mix into TOP10 cells (Invitrogen, Grand Island, NY) by electroporation, as recommended by the manufacturer. We grew the transformation mix on LB +50 µg/ml kanamycin at 37°C overnight. We picked colonies and transferred to 2 ml of LB liquid +50 µg/ml kanamycin to grow overnight in a 37°C shaking incubator. We isolated plasmid DNA from each culture using a miniprep kit (Qiagen, Hilden, Germany). We digested plasmid DNA with specific enzymes to verify the correct insertion. We recombined the *gipA* C_2_H_2_ region into pDEST 15 using an LR recombination reaction (Invitrogen, Grand Island, NY). We transformed the reaction mix into TOP10 cells (Invitrogen, Grand Island, NY) by electroporation, as recommended by the manufacturer. We grew the transformation mix on LB +100 µg/ml ampicillin at 37°C overnight. We picked colonies and transferred to 2 ml of LB liquid +100 µg/ml ampicillin to grow overnight in a 37°C shaking incubator. We isolated plasmid DNA from each culture using a miniprep kit (Qiagen, Hilden, Germany). We digested plasmid DNA with specific enzymes to verify the correct insertion. This vector, pDEST 15-gipA C_2_H_2_, was used in a protein binding microarray analysis as previously described [69].

### Mutagenesis of the GipA DNA Binding Site in the *gliA* Promoter

We created mutated *gipA* DNA binding sites in pDHGL using a QuikChange II XL Site-Directed Mutagenesis Kit (Agilent Technologies Inc., Santa Clara, CA), as recommended by the manufacturer. We utilized primers BSM1 F and BSM1 R, BSM2 F and BSM2 R to create vectors pDHBSM1 and pDHBSM2, respectively. We transformed pDHGL, pDHBSM1 and pDHBSM2 into Af293.1, as previously described for Af293.1-GL, except we plated transformants on YAG supplemented with uridine and uracil (described above), 0.2 M sucrose, and 300 µg/ml hygromycin. We verified correct transformants by Southern hybridization using the *lacZ* coding region as a probe (Fig. S8). We designated these strains as Af293.1-GL, Af293.1-BSM1 and Af293.1-BSM2, respectively. We obtained two independent isolates for each binding site mutant to verify that positional effects were not contributing to our results. To test the effect of the different *gipA* binding site mutants, we transformed pDONR AMA, pDONR AMA-*gliZ*, and pDONR AMA-*gipA* into two independent isolates of Af293.1-GL, Af293.1-BSM1, and Af293.1-BSM2, as described above for AMA-gipA.GL, except we grew transformants on YAG supplemented with 0.2 M sucrose. We designated these strains as AMA.GL, AMA-gliZ.GL, AMA-gipA.GL, AMA.BSM1, AMA-gliZ.BSM1, AMA-gipA.BSM1, AMA.BSM2, AMA-gliZ.BSM2, and AMA-gipA.BSM2.

### β-Galactosidase Assays

We ground 50 µl lyophilized mycelia to a fine powder with acid-washed glass beads (400–650 µm) (Sigma-Aldrich Corp., St. Louis, MO). We suspended the ground powder in 200 µl protein extraction buffer (PEB) (60 mM Na_2_HPO_4_·7H_2_O, 40 mM NaH_2_PO_4_·H_2_O, 10 mM KCl, 1 mM MgSO_4_·7H_2_O, 1 mM EDTA, and 20 µM PMSF [added fresh], pH 7.0) by vortexing and incubated the samples on ice for 15 min, with additional vortexing every 5 min. We spun tubes for 15 min at 15,600× g at 4°C to pellet cellular debris and beads. We transferred supernatants, containing total protein, to fresh tubes on ice and measured protein concentration using a Bio-Rad protein assay kit (Bio-Rad, Hercules, CA). In a 96-well plate, we added 10 µl of protein in PEB and 90 µl of Z Buffer (60 mM Na_2_HPO_4_·7H_2_O, 40 mM NaH_2_PO_4_·H_2_O, 10 mM KCl, 1 mM MgSO_4_·7H_2_O, 50 mM β-mercaptoethanol [added fresh], pH to 7.0). For samples grown in repressing conditions (CM), the total protein added was 1 µg. For samples grown in non-repressing conditions (CD), the total protein added was 0.1 µg. To begin the β-galactosidase assay, we added 20 µl of 2-Nitrophenyl-β-D-galactopyranoside (ONPG) (Sigma-Aldrich Corp., St. Louis, MO), diluted to 4 mg/ml in Z Buffer, and placed the 96-well plate in a 37°C incubator. We timed reactions and stopped samples with 50 µl 1 M Na_2_CO_3_. We measured absorbance at OD420 and calculated Units of β-galactosidase activity/mg protein with the following equation: (OD420×TV)/(0.0045×T×V×C), where TV is total volume of the reaction in ml, T is time in min, V is volume of protein added in ml, and C is concentration of protein used in µg/µl.

### Creation of Strains for Bypass Suppression Experiments

We grew Af293.1 in MAG supplemented with uridine and uracil (described above). We transformed pDEST R4-R3-*gliZ* 5′ FR-*AnpyrG*-*gliZ* 3′ FR and pDEST-*gipA* 5′ FR-*AnpyrG*-*gipA* 3′ FR into Af293.1, as previously described for *ΔgipA*, except we plated transformants on YAG supplemented with 0.2 M sucrose. We used *gliZ* 5′ and 3′ FRs and *gipA* 5′ and 3′ FRs for Southern hybridization and designated these strains *ΔgliZ.1* and *ΔgipA.1* (Fig. S9). We chose one strain from the *ΔgliZ* transformation that did not show homologous recombination at the *gliZ* locus, but was prototrophic for uridine and uracil. We designated this strain pyrG+. We collected total RNA and performed dot blot analysis on pyrG+, *ΔgliZ.1*, and *ΔgipA.1* to verify loss of *gliZ* and *gipA*, respectively (described in RNA dot blot Analysis) (Fig. S10). We cloned *gliZ* and *gipA* into pDONR AMA/HPH [63], as described above for pDONR AMA-*gliZ* and pDONR AMA-*gipA*, respectively, except we used primers gipA 3 kb F and 6g01910 R for the *gipA* cassette. We designated these plasmids pDONR AMA/HPH-*gliZ* and pDONR/HPH-*gipA*. We transformed these plasmids, along with pDONR AMA/HPH empty vector, into pyrG+, *ΔgliZ.1*, and *ΔgipA.1*, as described above for AMA-gipA.GL, except we grew transformants on YAG supplemented with uridine and uracil, 0.2 M sucrose, and 400 µg/ml hygromycin. We designated these strains as AMA.G, AMA-gliZ.G, AMA-gipA.G, AMA.Z, AMA-gliZ.Z, AMA-gipA.Z, AMA.A, AMA-gliZ.A, and AMA-gipA.A.

## Supporting Information

Figure S1 Complete microarray data. All genes tested are listed by locus. The average Log2 value from four independent experiments is shown for each locus listed.
(XLSM)

Figure S2 Secondary metabolism cluster graphs. Graphs depict Log2 values based on microarray data. Data are presented as mean ± SD. Clusters 1–18 (graphs 1–5) all contain at least one gene that is induced >2-fold in the presence of high-copy *gipA* expression. Clusters 19–31 (graphs 6–8) do not contain any genes that are induced >2-fold in the presence of high-copy *gipA* expression.
(TIF)

Figure S3 Southern hybridization of Af293.1-GL transformants. Genomic DNA was digested with EcoRI overnight at 37°C then run on a 0.8% agarose gel. The *lacZ* coding region was used as the probe. WT is wild-type (Af293.1) and is expected to have no band. Proper transformants should only have one band of unknown size. Lanes with asterisks are transformants that produced blue pigment when grown on MMVSN U/U (non-repressing) medium with X-gal.
(TIF)

Figure S4 Southern hybridizations of *gipA* and *gliZ* deletion mutants. Samples were run on a 0.8% agarose gel. WT is wild-type (Af1160). (a) *ΔgipA* transformants digested with PstI. WT should have a 4769 bp band and a 3275 bp band, while correct transformants are expected to have one 8627 bp band. The *gipA* 5′ FR and 3′ FR was used as a probe. (b) *ΔgliZ* transformants digested with KpnI. WT should have one 8775 bp band, while correct transformant should have a 6314 bp band and a 2945 bp band. The *gliZ* 5′ FR and 3′ FR was used as a probe.
(TIF)

Figure S5 Southern hybridization of *gipA(R)* transformants. WT is wild-type (Af1160). *gipA(R)* transformants were digested with SphI. WT should have two bands, one at 4131 bps and one at 3404 bps (the lower band is extremely light). The *ΔgipA* mutant (ΔA) should not display any band. Correct transformants should display two bands at 3404 bps and 1967 bps, which indicates 5′ integration of the complement plasmid. The *gipA* coding region was used as a probe.
(TIF)

Figure S6 Southern hybridization of *ΔgipA* mutants treated with 5-FOA. Samples were run on a 0.8% agarose gel. WT is wild-type (Af1160) and ΔA is the untreated deletion strain. *ΔgipA.0* mutants were digested with EcoRV and probed with the *gipA* coding region. Only WT should display a band, which is 7616 bp.
(TIF)

Figure S7 Southern hybridization of *ΔgliZ/ΔgipA* double mutants. Samples were run on a 0.8% agarose gel. WT is wild-type (Af1160). *ΔgliZ/ΔgipA* transformants were digested with SacI and probed with the *gliZ* and *gipA* coding regions. WT should display two bands, which are 7474 bps (*gipA*), and 4566 bps (*gliZ*). The *ΔgipA* control (ΔA) should display one band, which is 4566 bps (*gliZ*). Correct transformants should display not display the 7474 bp band or the 4566 bp band, signifying that the *gipA* and *gliZ* coding regions are not present, respectively. There is a 11712 bp band present in all lanes as a result of a third probe, but is not being mentioned here because it is not included in this paper.
(TIF)

Figure S8 Southern hybridizations of promoter mutagenesis transformants. Samples were digested with EcoRI and run on a 0.8% agarose gel. WT is wild-type (Af293.1) and GL is Af293.1-GL, which contains the *gliA-lacZ* construct. The *lacZ* coding region was used as the probe. WT should not display a band and GL should have a single band ∼6500 bps. Correct transformants should display a single band, any size. (a) First group of Af293.1-BSM1 transformants. Isolate 5 was chosen for experiments. (b) First group of Af293.1-BSM2 transformants. Isolate 4 was chosen for experiments. (c) Second group of Af293.1-GL and Af293.1-BSM1 transformants. Isolate 6 and 4 were chosen for experiments, respectively. (d) Second group of Af293.1-BSM2 transformants. Isolate 6 was chosen for experiments. Notice that isolates chosen for independent experiments for Af293.1-BSM1 and Af293.1-BSM2 display different sized bands, indicating that the pDHBSM1 and pDHBSM2 plasmids integrated in different places in the genome, respectively.
(TIF)

Figure S9 Southern hybridizations of *ΔgliZ.1* and *ΔgipA.1* transformants. Samples were run on a 0.8% agarose gel. WT is wild-type (Af293.1). (a) *ΔgliZ.1* transformants were digested with KpnI. WT should display a 8775 bp band and correct transformants should display two bands at 6314 bps and 2945 bps. The *gliZ* 5′ and 3′ FRs were used as a probe. (b) *ΔgipA.1* transformants were digested with PstI. WT should display two bands at 4769 bps and 3275 bps, while correct transformants should display one band at 8627 bps. The *gipA* 5′ and 3′ FRs were used as a probe.
(TIF)

Figure S10 Verification of *ΔgliZ.1* and *ΔgipA.1* mutants in an Af293.1 background. (a) Cultures were grown in non-repressing conditions at 37°C for 48 hrs. Total RNA was collected and dot blot analysis was performed in triplicate with 3 µg RNA/spot. RNA levels are relative to pyrG+. The results of one representative experiment of two biological replicates are shown. The asterisk (*) indicates a statistically significant difference (p-value<0.05), compared to PyrG+, calculated by one-way ANOVA and Tukey comparison test. (b) Gliotoxin was measured with RP-HPLC.
(TIF)

Table S1 Primers used in this study. Bolded regions are *att* sites. Underlined regions are extensions for fusion PCR. Uppercase regions are NotI sites.
(DOCX)

Table S2 Strains used in this study and genotypes.
(DOCX)
