# Supplementary figures and images for: A Novel C2H2 Transcription Factor that Regulates gliA Expression Interdependently with GliZ in Aspergillus fumigatus
Source: PLoS Genet. 2014 May 1;10(5):e1004336. doi: 10.1371/journal.pgen.1004336 (PMC4006717; doi:10.1371/journal.pgen.1004336)

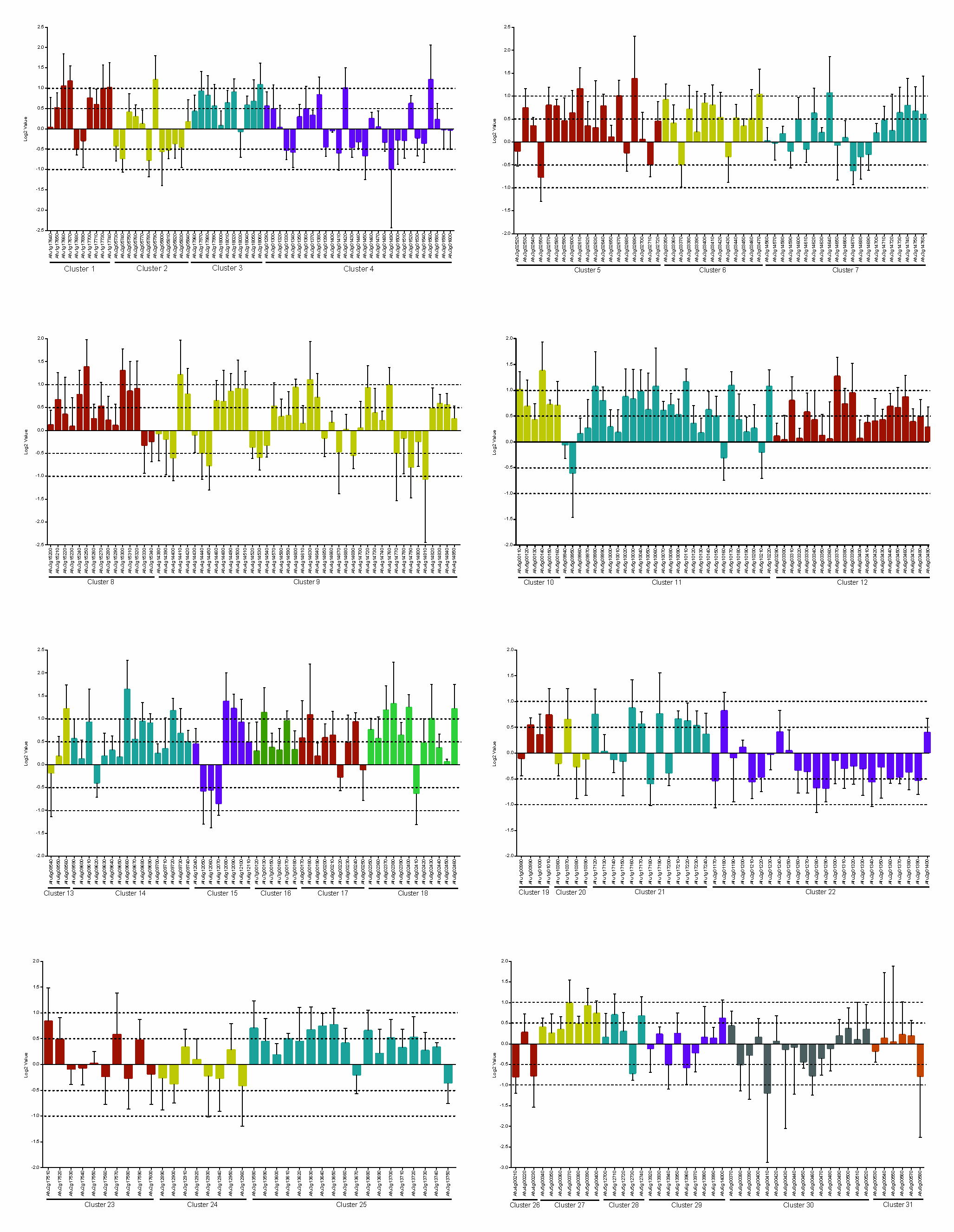

Supplement: Figure S2 — Secondary metabolism cluster graphs. Graphs depict Log2 values based on microarray data. Data are presented as mean ± SD. Clusters 1–18 (graphs 1–5) all contain at least one gene that is induced >2-fold in the presence of high-copy gipA expression. Clusters 19–31 (graphs 6–8) do not contain any genes that are induced >2-fold in the presence of high-copy gipA expression. (TIF) [file pgen.1004336.s002.tif]

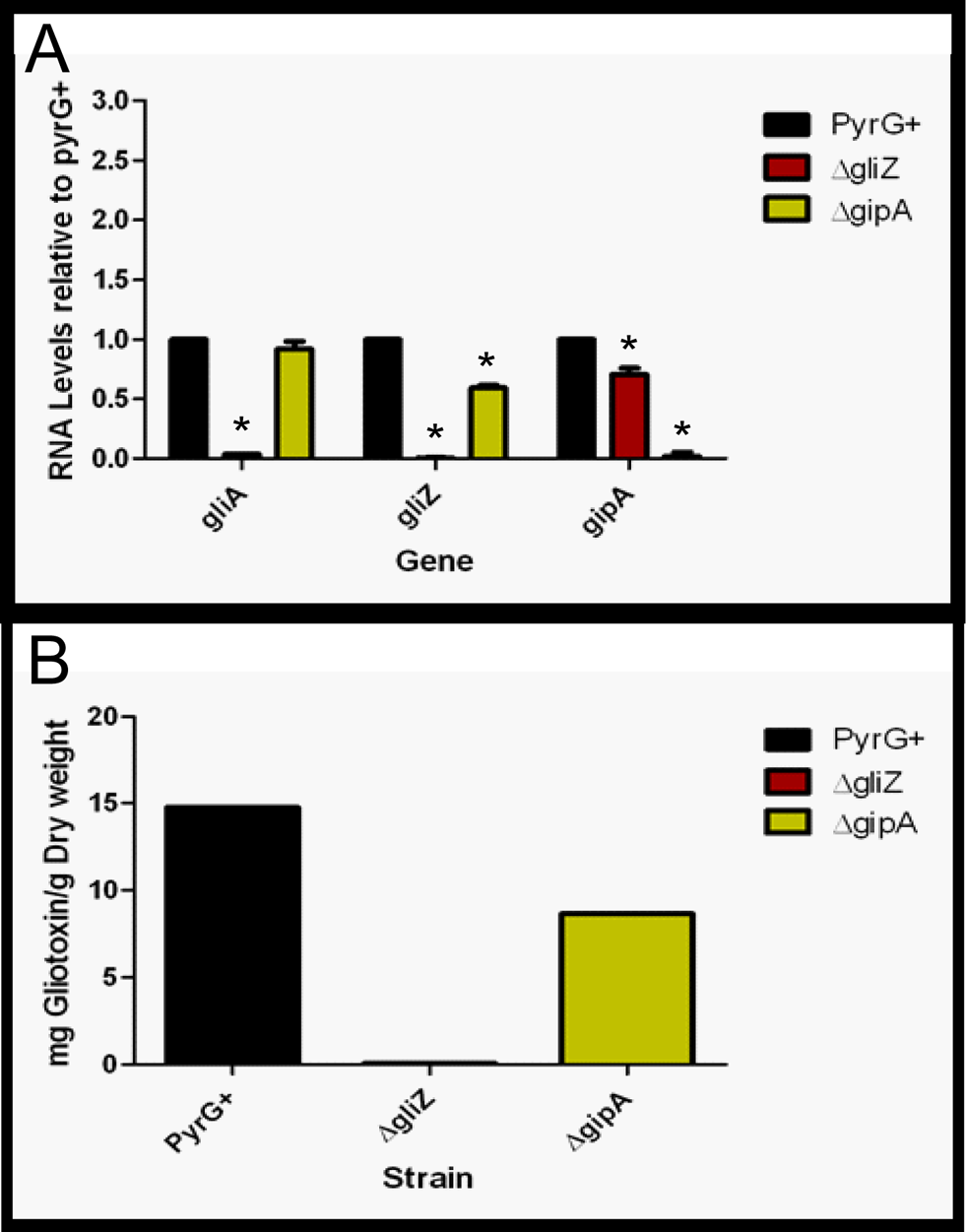

Supplement: Figure S10 — Verification of ΔgliZ.1 and ΔgipA.1 mutants in an Af293.1 background. (a) Cultures were grown in non-repressing conditions at 37°C for 48 hrs. Total RNA was collected and dot blot analysis was performed in triplicate with 3 µg RNA/spot. RNA levels are relative to pyrG+. The results of one representative experiment of two biological replicates are shown. The asterisk (*) indicates a statistically significant difference (p-value<0.05), compared to PyrG+, calculated by one-way ANOVA and Tukey comparison test. (b) Gliotoxin was measured with RP-HPLC. (TIF) [file pgen.1004336.s010.tif]
