# Supplementary material for: A Novel C2H2 Transcription Factor that Regulates gliA Expression Interdependently with GliZ in Aspergillus fumigatus
Source: PLoS Genet. 2014 May 1;10(5):e1004336. doi: 10.1371/journal.pgen.1004336 (PMC4006717; doi:10.1371/journal.pgen.1004336)
Supplement: Table S1 — Primers used in this study. Bolded regions are att sites. Underlined regions are extensions for fusion PCR. Uppercase regions are NotI sites. (DOCX) [file pgen.1004336.s011.docx]

| **Name** | **Sequence** |
| --- | --- |
| GliA F1 | 5’-**ggggacaagtttgtacaaaaaagcaggctaa**gatagcaacagtagccaatgt-3’ |
| GliA 5’ R | 5’-cgtaatcatggtcataatggtcgatgtcagtagagagctg-3’ |
| lacZ F | 5’-ctgacatcgaccattatgaccatgattacggattcactgg-3’ |
| lacZ R | 5’-gtttcgaccagatacttatttttgacaccagaccaactgg-3’ |
| GliA 3’ F | 5’-tggtgtcaaaaataagtatctggtcgaaacatgtctgctt-3’ |
| GliA R | 5’-**ggggaccactttgtacaagaaagctgggtc**taagctcgggatggagtgatt-3’ |
| GliZ attB 1 | 5’-**ggggacaagtttgtacaaaaaagcaggctgc**gaccgcagctgattggag-3’ |
| GliZ attB 2 | 5’-**ggggaccactttgtacaagaaagctgggtc**gattccctttgtgccgcc-3’ |
| AMA-NotI F | 5’-aaataagcttgcatgcgc-3’ |
| AMA-NotI R | 5’-gccagtgaattcgagctc-3’ |
| 6g01910 F | 5’-**ggggacaagtttgtacaaaaaagcaggctg**cgggtttggtttggttgttgtgctt-3’ |
| 6g01910 R | 5’-**ggggaccactttgtacaagaaagctgggtc**gagggcgtggtgaacgttc-3’ |
| M13F | 5’-cgccagggttttcccagtcacgacg-3’ |
| M13R | 5’-ggaaacagctatgaccatga-3’ |
| 01910 5’ F | 5’-**ggggacaactttgtatagaaaagttgaa**GCGGCCGCgcttacttacagtacggagtacgg-3’ |
| 01910 5’ R | 5’-**ggggactgcttttttgtacaaacttgc**gcccggcggaggaat-3’ |
| 01910 3’ F | 5’-**ggggacagctttcttgtacaaagtggaa**tccgttttctacgagcattgttctc-3’ |
| 01910 3’ R | 5’-**ggggacaactttgtataataaagttgc**ttcatggtgccgtgctcg-3’ |
| gipA 3kb F | 5’-**ggggacaagtttgtacaaaaaagcaggctgc**accccgtttttgtggttgcgc-3’ |
| gliZ 5’ F | 5’-**ggggacaactttgtatagaaaagttgaa**GCGGCCGCgggagtcgagagatgcatgaa-3’ |
| gliZ 5’ R | 5’-**ggggactgcttttttgtacaaacttgc**tgtggatgtcggggacga-3’ |
| gliZ 3’ F | 5’-**ggggacagctttcttgtacaaagtggaa**gctgttctcacctcttttttttttttt-3’ |
| gliZ 3’ R | 5’-**ggggacaactttgtataataaagttgc**cgagctcgtcgaccagta-3’ |
| gipA C2H2 F | 5’-**ggggacaagtttgtacaaaaaagcaggctgc**cagcaaatgtacggcgggca-3’ |
| gipA C2H2 R | 5’-**ggggaccactttgtacaagaaagctgggtc**tcagctgtgcccattggtatcaacg-3’ |
| BSM1 F | 5’-gacttaacggagactctgccgccacgccgaatcacagcgg-3’ |
| BSM1 R | 5’-ccgctgtgattcggcgtggcggcagagtctccgttaagtc-3’ |
| BSM2 F | 5’-gacttaacggagactttgggtgagcgccgaatcacagcgg-3’ |
| BSM2 R | 5’-ccgctgtgattcggcgctcacccaaagtctccgttaagtc-3’ |
