# Supplementary material for: A Novel C2H2 Transcription Factor that Regulates gliA Expression Interdependently with GliZ in Aspergillus fumigatus
Source: PLoS Genet. 2014 May 1;10(5):e1004336. doi: 10.1371/journal.pgen.1004336 (PMC4006717; doi:10.1371/journal.pgen.1004336)
Supplement: Table S2 — Strains used in this study and genotypes. (DOCX) [file pgen.1004336.s012.docx]

| **Strain** | **Genotype** | **Origin** |
| --- | --- | --- |
| Af293 | Wild type | FGSC |
| Af293.1 | pyrG1 | This Lab[49] |
| Af293.1-GL | pyrG1; pDHGL | This Study |
| AMA.GL | pyrG1; pDHGL; pDONR AMA | This Study |
| AMA-gliZ.GL | pyrG1; pDHGL; pDONR AMA-gliZ | This Study |
| AMA-gipA.GL | pyrG1; pDHGL; pDONR AMA-gipA | This Study |
| 1160 | pyrG1; nkuB::AfpyrG; pyrG::5FOA | FGSC[83] |
| 1160G | pyrG1; nkuB::AfpyrG; pyrG::5FOA; pDONR G | This Lab |
| *ΔgliZ* | pyrG1; nkuB::AfpyrG; pyrG::5FOA; pDHGL; gliZ::pyrG | This Study |
| *ΔgipA* | pyrG1; nkuB::AfpyrG; pyrG::5FOA; gipA::pyrG | This Study |
| *gipA*(R) | pyrG1; nkuB::AfpyrG; pyrG::5FOA; gipA::pyrG; pDONR HPH-gipA | This Study |
| *ΔgipA.0* | pyrG1; nkuB::AfpyrG; pyrG::5FOA; gipA::pyrG; pyrG::5FOA | This Study |
| *ΔgliZ/ΔgipA* | pyrG1; nkuB::AfpyrG; pyrG::5FOA; gipA::pyrG; pyrG::5FOA; gliZ::pyrG | This Study |
| Af293.1-BSM1 | pyrG1; pDHBSM1 | This Study |
| Af293.1-BSM2 | pyrG1; pDHBSM2 | This Study |
| AMA.BSM1 | pyrG1; pDHBSM1; pDONR AMA | This Study |
| AMA-gliZ.BSM1 | pyrG1; pDHBSM1; pDONR AMA-gliZ | This Study |
| AMA-gipA.BSM1 | pyrG1; pDHBSM1; pDONR AMA-gipA | This Study |
| AMA.BSM2 | pyrG1; pDHBSM2; pDONR AMA | This Study |
| AMA-gliZ.BSM2 | pyrG1; pDHBSM2; pDONR AMA-gliZ | This Study |
| AMA-gipA.BSM2 | pyrG1; pDHBSM2; pDONR AMA-gipA | This Study |
| pyrG+ | pyrG1; AnpyrG | This Study |
| ΔgliZ.1 | pyrG1; gliZ::pyrG | This Study |
| ΔgipA.1 | pyrG1; gipA::pyrG | This Study |
| AMA.G | pyrG1; pyrG; pDONR AMA/HPH | This Study |
| AMA-gliZ.G | pyrG1; pyrG; pDONR AMA/HPH-gliZ | This Study |
| AMA-gipA.G | pyrG1; pyrG; pDONR AMA/HPH-gipA | This Study |
| AMA.Z | pyrG1; gliZ::pyrG; pDONR AMA/HPH | This Study |
| AMA-gliZ.Z | pyrG1; gliZ::pyrG; pDONR AMA/HPH-gliZ | This Study |
| AMA-gipA.Z | pyrG1; gliZ::pyrG; pDONR AMA/HPH-gipA | This Study |
| AMA.A | pyrG1; gipA::pyrG; pDONR AMA/HPH | This Study |
| AMA-gliZ.A | pyrG1; gipA::pyrG; pDONR AMA/HPH-gliZ | This Study |
| AMA-gipA.A | pyrG1; gipA::pyrG; pDONR AMA/HPH-gipA | This Study |
